# Assessment of Ecosystem Service Quality and Its Correlation with Landscape Patterns in Haidian District, Beijing

**DOI:** 10.3390/ijerph16071248

**Published:** 2019-04-08

**Authors:** Boya Wang, Zhicheng Liu, Yuting Mei, Wenjie Li

**Affiliations:** 1 Beijing Laboratory of Urban and Rural Ecological Environment, Beijing Municipal Education Commission, School of Landscape Architecture, Beijing Forestry University, 35 Tsinghua East Rd., Haidian District, Beijing 100083, China; boyawang@bjfu.edu.cn (B.W.); yutingmei19@gmail.com (Y.M.); 2 Orirent Landscape, IT Industrial Park, 104 Floor, 10 Courtyard Electronic City, Jiuxianqiao North Road, Chaoyang District, Beijing 100015, China; liwj0724@163.com

**Keywords:** ecosystem services quality, assessment, landscape pattern, correlation, optimization strategy, Haidian District

## Abstract

Landscape architecture with urban green space as the main research object is an evidence-based science. It is an important issue to optimize green space systems from the point of view of ecosystem services. In this paper, high-resolution (1.5 m resolution) remote sensing images are combined with data-processing software, such as ENVI, ArcGIS, and Fragstats, to evaluate ecosystem service quality and compute the landscape pattern in the Haidian District (Beijing, China), so that the relationship between the ecosystem service quality and landscape pattern can be quantitatively studied and a strategy can be provided for green space optimization in cities. The following conclusions are drawn: (1) for the evaluated quality of 14 ecosystem services in Haidian District (refer to Section Analysis of the Association of the Percentage of Patches (PLAND) Index of Forest Land and Quality of Ecosystem Service in Haidian District). Forest land is the main provider of the ecosystem service in Haidian District, while construction land only provides cultural services; (2) on the whole, the spatial distribution of the ecosystem services in Haidian District gradually decreases from the west to the east, which basically matches with the spatial distribution of the forest land. The regulating service and supporting service are matched with the distribution of the urban green space. The cultural service is closely associated with history resource points; and (3) the analysis results of the association between landscape pattern and ecosystem service quality show that the percentage of patches (PLAND) index for forest land has a significant logarithmic relationship with the regulating service and supporting service. The critical value of the PLAND index is 30. Besides the Xishan area with the most coverage of forest land, the landscape shape index (LSI) of the brushwood has a logarithmic relationship with the ecosystem service quality. The critical value of the LSI value is 50. Finally, this paper proposes an area optimization strategy of green space in Haidian District from the view of the ecosystem system service. The Xishan area is classified into the ecosystem red line to control city expansion. The regulating and supporting services can be enhanced in the north flat area by improving the patch shape index. The ecosystem service capabilities can be improved by adding the forest land in the existing green space for the southeast urban areas.

## 1. Introduction

Ecosystem services (ES) are considered to be the benefits obtained by human beings from an ecosystem. ES are generally divided into four categories: supply services, regulating services, supporting services, and cultural services [1]. As a bridge between human beings and nature, ecosystem services have attracted more and more attention from urban construction scholars, especially for the construction of urban green space dominated by both natural attributes and human factors.

In this study, the “China National Knowledge Internet” and “Web of Science” were searched using “greenspace* ecosystem service*” as keywords, and a total 157 references were retrieved. These research works mainly focus on research progress of ecosystem service functions [2,3,4,5,6,7,8,9,10,11,12], the association between ecosystem services and landform evolution [13,14,15,16,17,18,19,20], and evaluation method of ecosystem services [21,22,23,24,25,26,27,28,29,30,31,32,33,34,35,36,37,38,39,40,41,42,43,44,45,46,47,48]. Meanwhile, research on applications in urban construction and practices is scarce. In the evaluation method and application, Burley proposed the multi-model habitat suitability procedure in landscape planning, then from the micro and macro level did some experiments. For example, nineteen species were studied to proposed landscape modifications and configurations in the Red River Valley, then prioritize protection levels [49]; through the impact of soil characteristics on different plant productivity in post-mining landscape configuration in Clay County, Burley addresses the development of a vegetation productivity equation for predicting plant growth potential, which provided specific guidance with landscape planning [50]. Loures focus his research on the built environment and its artificial components. Greenspace in city is recognized as strategic planning elements for achieving sustainable development, which is extremely important for citizens’ quality of life, contributing to increase land value and sustainable city development [51]. In term of research in Beijing, Cui and Xu proposed the concept of “Ideal Forest Land Equivalent” to evaluate ecosystem services of green spaces in Beijing City and give corresponding enhancement strategies for urban construction [52]. Feng studied layout optimization of agricultural lands in the flat areas of Beijing City by using the ecosystem service value [53].

In recent years, affected by urbanization, urban green spaces have been encroached on massively, so that the area of green space has decreased quickly, is of low quality, and has a chaotic layout. Consequently, the urban ecological environment is threatened and the ecosystem service is also severely affected. Therefore, quality improvement and layout optimization have become main research topics for the improvement of ecosystem services under the prerequisite of no growth of green space. This paper tries to divide research units in Haidian District and study the relationship between the ecosystem service quality and landscape pattern, in order to provide a basis for urban green space system optimization from the ecological point of view and provide feasible strategies for the improvement of urban ecosystem.

The United Nations Millennium Ecosystem Assessment (MA) regulates that the ecosystem services include supply service, regulating service, cultural service, and supporting service. Haidian District is located in the central area of Beijing City, China, and mainly depends on external systems to provide resources such as substances and energy, and does not supply substances by its self. Therefore, the supply service of substances and resources such as food and water is not regarded as the main content of the green space. This paper mainly studies the regulating service, supporting service, and cultural service of Haidian District. 

The regulating service involves regulating the human ecological environment, including the regulation and control of floods, droughts, land deterioration, diseases, carbon fixation and oxygen release, cooling and humidifying, air purification, and water conservation. The carbon fixation and oxygen release service is implemented based on two principles. The first principle is to absorb and fix the CO_2_ via plant photosynthesis and growth function. The second principle is to reduce the CO_2_ release from fossil fuels by direct CO_2_ absorption from plants and soils or tree shade and evaporation [54]. Cooling and humidifying are indicated in terms of two aspects. The first aspect is the tree shadow. The research results show that 20–50% of heat will be reflected to the air and 35% of heat is absorbed by tree crowns when sunlight is radiated to crowns. The second aspect is evaporation from green lands. When water is vaporized, it absorbs much energy [55]. The air is purified mainly via absorption of plants, including chemical absorption and physical absorption. Some harmful gases, such as sulfur dioxide, oxynitride, and hydrogen fluoride will mainly form organic materials via chemical action. Some solid particles, such as dust and powder, are mainly blocked, filtered, and absorbed by leaves, especially plants with seta, fluffy, and coarse leaf surface, and plants with oil and grime on the leaf surface have better effect [56]. Urban green spaces intercept rainfall, suppress evaporation, reduce surface runoff, and increase the underground runoff to conserve contents under common action of plants, soil, and weather, and the water conservation capabilities can be quantified by regulating water capacity [57]. 

The supporting service is an elementary function required to ensure other ecosystem service functions, and it can influence human beings in an indirect or long-term action, e.g., soil formation, nutrient circulation, and soil conservation. The soil conservation is based on the following principle: The living mulch and withered and fallen substance layer will intercept rainfall to reduce the flushing of water drops on surface soils and corrosion of surface runoff. The plant root system can conserve soils, avoid the collapse and effusion of soils, reduce soil fertility loss, and improve soil structure [58]. 

Cultural service indicates intangible interests, entertainment, spiritual feeling, and aesthetic experience acquired by people from the ecosystem. Cultural services are very subjective and are difficult to evaluate quantitatively. With the development of technology and the generation of big data, partial experts and scholars evaluate ecosystem cultural services by using the point of interest (POI) of photos. 

## 2. Materials and Methods 

### 2.1. Haidian District Overview

**Figure 1:**
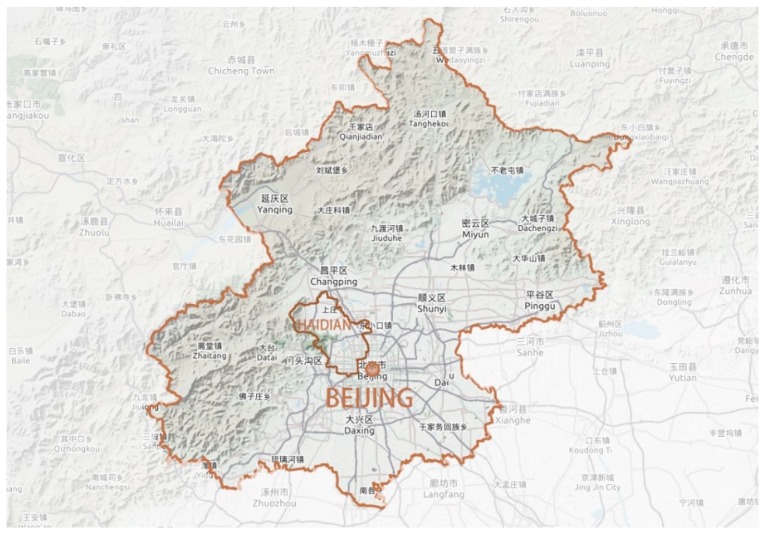
Map showing the location of Haidian District.

The Haidian District is located in the northwest area of the central area of Beijing City (Figure 1) and has an area of 430.8 km^2^. The Haidian District has a semi-humid continental monsoon climate of a warm temperate zone. The average annual rainfall is 585 mm. The district is located at the crossing point between the edge of the northern North China Plain and the Taihang Mountains and includes the mountainous areas in the west with over 100 m elevation and the east plain. Haidian District includes about 10 large and small rivers, and the green coverage is 52.2%. The Haidian District is located at the combination belt of a shallow mountainous area, plain area, and urban constructed are, and has rich green space and a diversified landscape pattern. This district has historical cultural resources, with “Three Hills and Five Parks” as the core, and has very high ecological, cultural, and social value. 

### 2.2. Method

**Figure 2:**
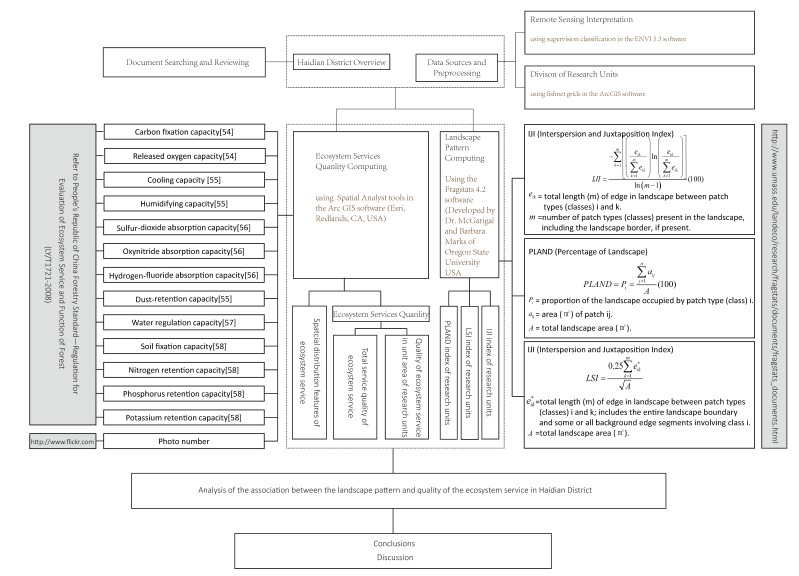
The technical route on the study.

A variety of technical methods and means were adopted in the study, and the specific technical recording route is shown in Figure 2.

#### 2.2.1. Data Sources and Processing

**Figure 3:**
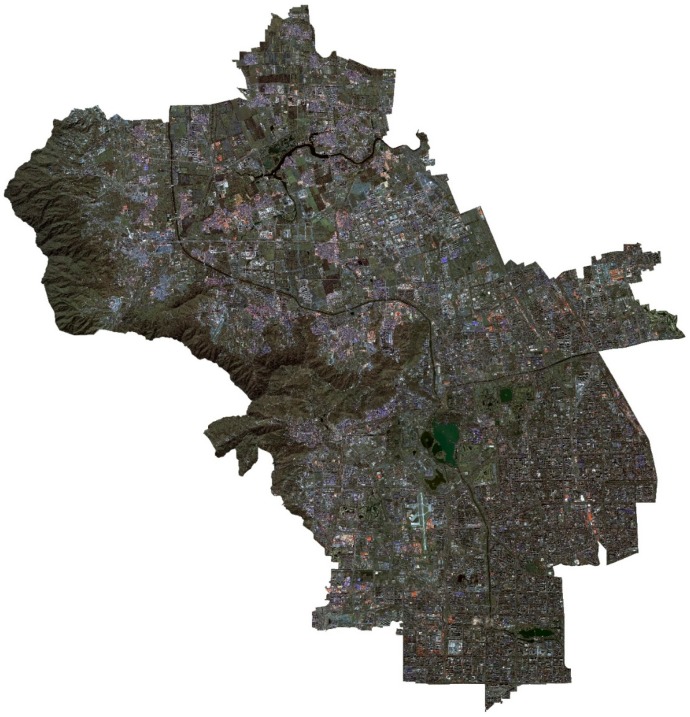
The high-resolution (1.5 m resolution) remote sensing image of Haidian District obtained by the GF-2 satellite.

A high-resolution (1.5 m resolution) remote sensing image of the GF-2 satellite taken on 12 September 2015 was used as the data source (images purchased from the China Resources Satellite Application Center were processed by the Beijing Changdi Friends Mapping Technology Center). Geometric correction, radiometric calibration, and atmospheric correction were performed in pre-processing (Figure 3). The supervision classification in the ENVI 5.3 software (Harris Geospatial Solutions, Broomfield, CO, USA) is used to explain green space in Haidian District. With Classification of Land Utilization Conditions as the classification reference, and by combining actual conditions in the research area, the green space in Haidian District can be divided into forest land, brush, grassland, water area, construction land, and other lands. The explanation standards are shown in Table 1.

#### 2.2.2. Division of Research Units

**Figure 4:**
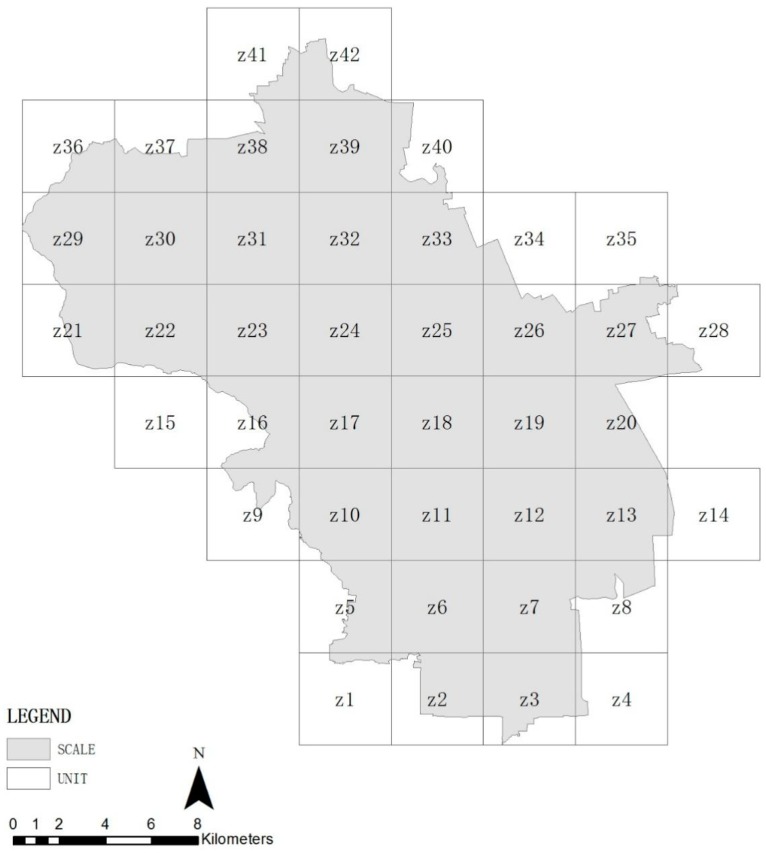
Research units in Haidian District.

In order to study the relationship between the ecosystem service quality and landscape pattern in Haidian District, the picture was evenly divided into 4000 × 4000 m fishnet grids in the ArcGIS software (ESRI, Redlands, CA, USA), and a total of 42 research units were obtained (Figure 4) to study the relationship between the ecosystem service quality and landscape pattern. Compared to the administrative area division, terrain, landform, and urbanization, the grid division can eliminate influences such as man-made management and natural features, and data results are not objective.

#### 2.2.3. Computing Method of Ecosystem Service Quality

The service quality evaluation method is a quantitative method that is frequently used for the analysis of ecosystem service, and which can objectively show structures, functions, and ecology of the ecosystem. In this paper, the carbon fixation and oxygen release, cooling and humidifying, air purification, water conservation, soil fixation and fertility conservation functions are selected, according to forest ecosystem service function evaluation specification (LY/T1721-2008) issued by the State Forestry Bureau, by combining related research work of Wu [59]. The service quality evaluation index system of the ecosystem service in Haidian District is constructed according to actual data from Beijing City. 

The cultural services are expressed by using the number of POI photo with coordinate information. Flickr (SmugMug, San Francisco, CA, USA) is the largest image sharing website in the world and includes detailed geological information and text labels. Wang [60] analyzed the attraction degree of the landscape based on the Flickr website. Luo identified the scenes based on the Flickr website [61]. Xue [62] evaluated the terrain of a tourist destination based on the Flickr website. For the photographic data analyzed in this paper, the photo number is used to express the cultural service capabilities. The specific steps are as follows: download images with geological coordinate information by using the Flickr Application Programming Interface (API); select labels such as “outdoor” and “landscape”; remove unrelated photos; and, finally, obtain 3363 scene photos from Haidian District, Beijing City, and express the cultural service capabilities by using the number of photos.

#### 2.2.4. Computing Method of Landscape Pattern 

The Fragstats 4.2 software (Oregon State University, Corvallis, OR, USA) was used to analyze the landscape pattern based on the interpreted data. According to the research results of Cen [63], three landform indices of the patch type level are selected for analysis, including percentage of landscape (PLAND) index, interspersion and juxtaposition index (IJI), and landscape shape index (LSI), for forest land. The PLAND index indicates the percentage of the patch in the landscape and describes the quality characteristics of the research area. The IJI index is used to express the general distribution of the landscape. A higher value indicates that the alternate occurrence law of different patches is more significant, and the types are distributed. The LSI shows the complexity of the shape.

## 3. Results

### 3.1. Quality of Ecosystem Services in Haidian District

#### 3.1.1. Total Service Quality of Ecosystem Service 

**Figure 5:**
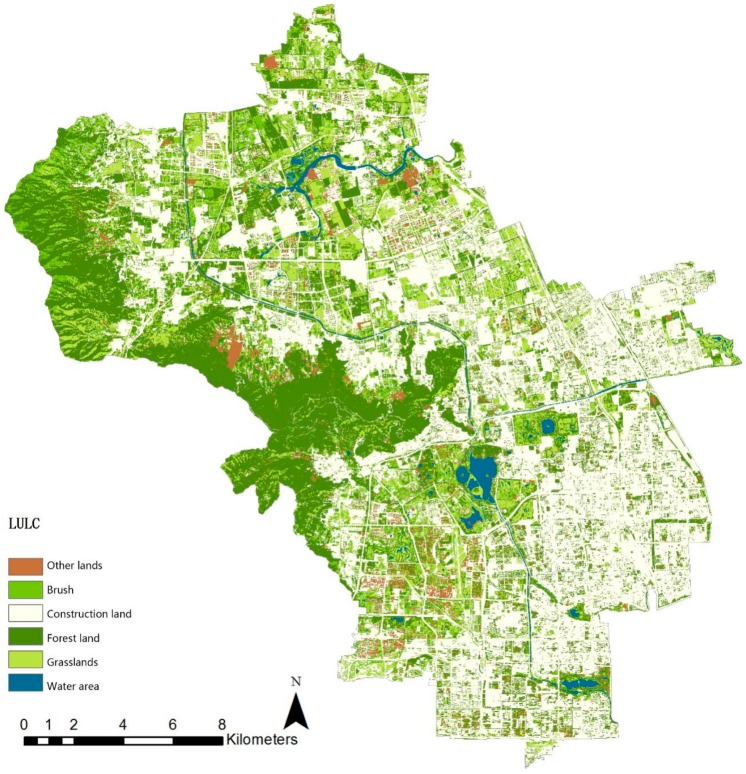
The green space interpretation for Haidian District.

The land coverage data for Haidian District in 2015 (Figure 5), whose interpretation precision is over 80%, shows that the total area of the forest land, brush, grassland, water, and other lands is 23.7 km^2^, which is 56% of the total area of Haidian District. The area of forest land is 12 km^2^, which is half the total area of green space. The area of brush and grassland is basically the same, and the percentage of water is smaller. On the whole, the total green space in Haidian District is rich and mainly includes forest land. 

After calculation, the total quality of the ecosystem service in Haidian District in 2015 is described as follows: For regulating service, the carbon fixation capacity and oxygen release capacity are 92, 339, 108 kg, and 247, 206, 505 kg, respectively. The cooling capacity and humidifying capacity are 1, 178, 705, 379, and 339 KJ, and 417, 718, 842, and 3 kg, respectively. The sulfur dioxide, oxynitride, and hydrogen fluoride capacity of the purified air are 2, 368, 389, and 597 kg, 129, 312, and 450 kg, and 4, 19, and 51 kg, respectively. The stagnant dust capacity is 313, 202, 416, and 986 kg. The regulation water capacity is 14, 544, 419, and 439 kg. For the supporting service, the soil fixation capacity is 614, 729, and 832 kg. The nitrogen, phosphor and potassium retention capacity in the soil conservation are 101, 430, and 422 kg, 7, 991, and 488 kg, and 88, 521, and 96 kg, respectively. For the cultural service, total photo is 3, 363. The quality for the ecosystem services of different land coverage types are shown in Table 2. Forest land is the main provider of the ecosystem service in Haidian District, and the quality of its services is over half. The construction land only provides cultural service. 

#### 3.1.2. Quality of Ecosystem Service in Unit Area of Research Units 

**Figure 6:**
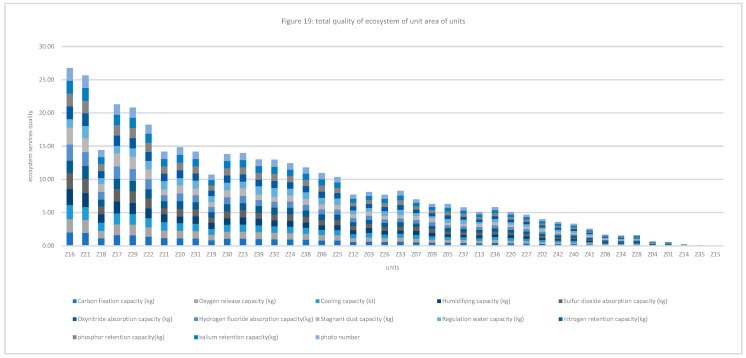
Total quality of ecosystem of areas of units.

The quality of the ecosystem service of unit area of 42 cells was calculated, and compared with the quality per unit area in Haidian District. The results are shown in Table 3. For the regulating service, the quality of the ecosystem service of units Z10, Z16, Z17, Z18, Z21, Z22, Z23, Z29, and Z31 are more than the average in Haidian District. Except for the regulating water capacity, the quality of units Z18 and Z23 is more than the average. Except for the humidifying capacity, the quality of unit Z30 is more than the whole average. The sulfur dioxide, hydrogen fluoride, and stagnant dust capacity absorbed by units Z11 and Z39 are lower than the average. The water regulation capacity of units Z24 and Z38 is more than the average, which indicates stronger water conservation capability. The quality of the supporting services of units Z10, Z11, Z16, Z17, Z18, Z21, Z22, Z23, Z29, Z30, Z31, Z32, and Z39 is better than the average in Haidian District. The “Three Hills and Five Parks”, important historical cultural resources in Haidian District, are located in units Z11, Z17, Z18, and Z19, and the cultural service capabilities of these four units are far stronger than those of other areas; therefore, the cultural service is closely associated with the historical resource points The ratio of the quality of the units to the average is used for data standardization in research. The total quality of the ecosystem of the area of units is shown in Figure 6. The quality of units Z10, Z11, Z16, Z17, Z18, Z19, Z21, Z22, Z29, and Z31 is higher than the average. The quality of unit Z30 is close to the average of Haidian District. The percentages of the forest land, brush, grassland, water area, construction land, and other lands are 26%, 17%, 12%, 1%, 42%, and 3%, respectively. The quality of the ecosystem service of units Z16 and Z21 is maximal, and the percentage of the forest land in these units is 83% and 61%, respectively. 

### 3.2. Spatial Distribution Features of Ecosystem Service in Haidian District

**Figure 7:**
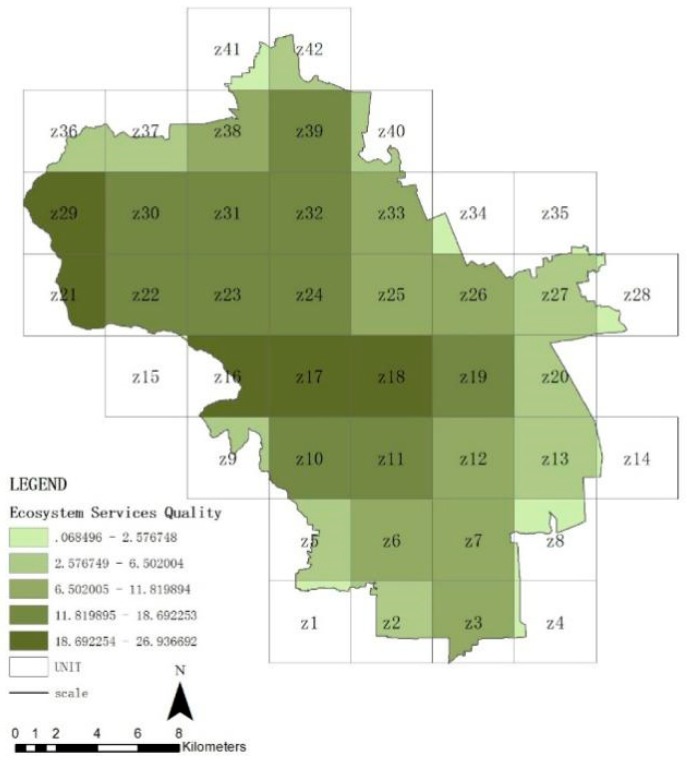
Quality distribution of ecosystem service of units.

**Figure 8:**
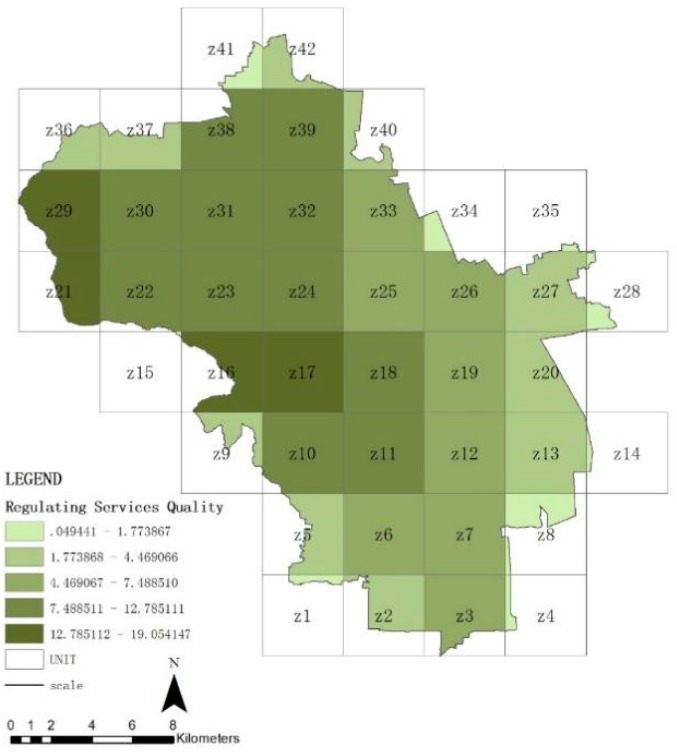
Quality distribution of regulating service of units.

**Figure 9:**
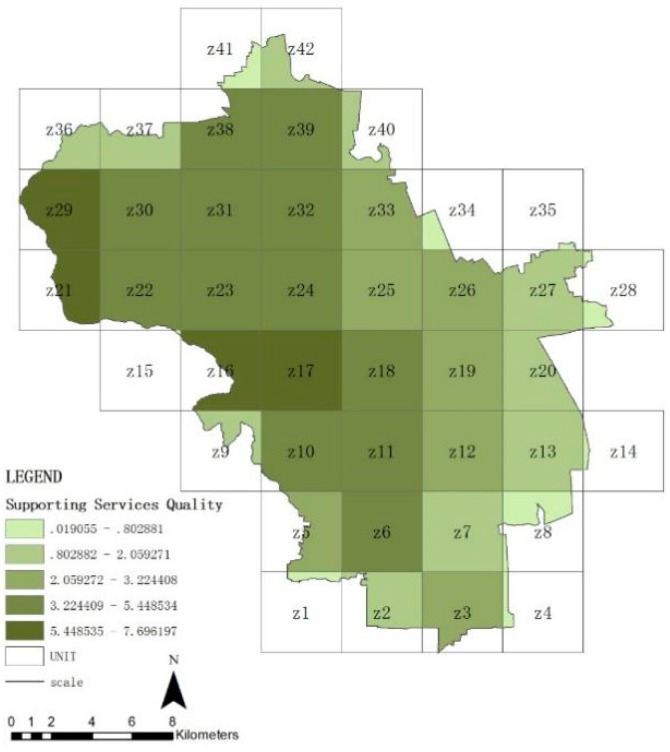
Quality distribution of supporting service of units.

**Figure 10:**
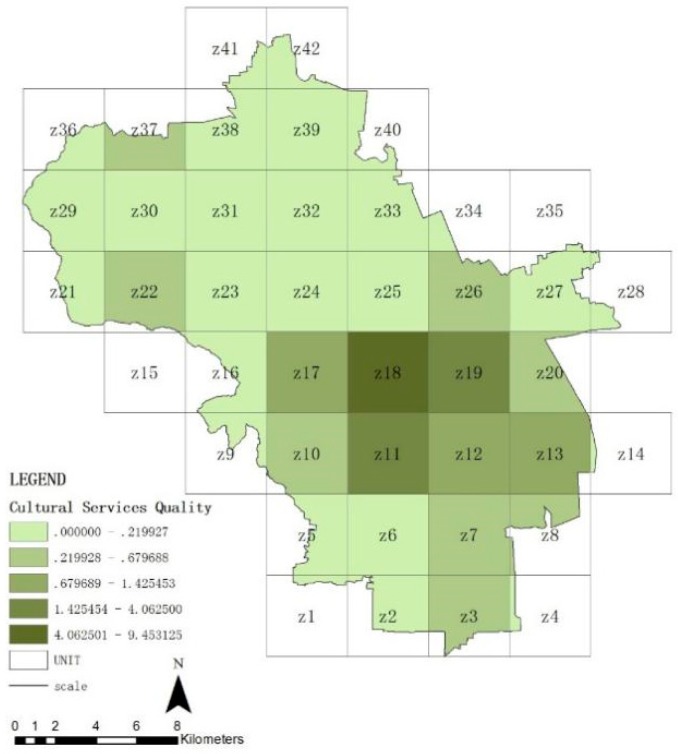
Quality distribution of cultural service of units.

Figure 7, Figure 8, Figure 9 and Figure 10 show the quality of the ecosystem service of units after standardization and is divided into five levels by the natural point break method. On the whole (Figure 7), the ecosystem service of Haidian District gradually decreases from the west to the east, and the values of the northwestern units Z16, Z17, Z18, Z21, and Z29 are maximum, which match with the distribution of forest land. The quality of the northern plain (units Z22, Z23, Z24, Z30, Z31, Z32, and Z39) and the southwestern area ahead of hills (units Z10, Z11, and Z19) is followed. The whole share of the green space in these areas is higher. The quality of the ecosystem in the southeastern urban areas is minimal, which is closely related with the share of construction lands, i.e., the urban construction strength. From these data, forest land can be seen to be the significant positive influencing factor for the quality of the ecosystem service in Haidian District, and construction land to be the main negative influencing factor. The quality of the regulating service (Figure 8) and supporting service (Figure 9) is maximum in the western mountainous area, followed by the northern plain. The quality of the southeastern urban area is minimal, and is basically matched with the distribution of the urban green space. This indicates that the green space area (including forest land, brush, grassland, and water area) is the main factor affecting the regulating service and support service. The cultural service (Figure 10) of “Three Hills and Five Parks” (Z18) is optimal. The cultural service of the southeastern urban area is better than that of the western mountainous area. This indicates that the cultural service is closely associated with the quality and features of the green space. The very high cultural service capability of unit Z18 can significantly improve the quality of the whole ecosystem service. 

### 3.3. Analysis of the Association between the Landscape Pattern and Quality of the Ecosystem Service in Haidian District

The Fragstats software was used to calculate the landscape indices of 42 research units, and the calculated results are shown in Table 4. For the PLAND index, the values of the construction land of units Z13, Z7, Z12, and Z27 are maximal, and are 76.87, 72.26, 69.71, and 65.63, respectively, while the maximal index values of the forest land in units Z17 and Z15 is 61.14 and 58.07, respectively. The LSI values of the brush in units Z11, Z6, Z39, Z30, Z10, and Z33 are maximal, and are 76.23, 75.63, 72.93, 72.39, 71.07, and 70.77, respectively. The IJI index values of the water area in unit Z09 and of other lands in unit Z12 are maximal, and are 89.73 and 86.48. The above data shows that the PLAND index features of the forest lands and construction lands in research units are significant and the percentage of the forest lands is very different from that of the construction lands in units. The LSI features of the brush are significant, which indicates that the shapes of the brush patches are the most complicated in the research units. The LJI indices of water areas and other lands are significant, which indicates that the water areas and grasslands are distributed sparsely in the research units. 

The association between the landscape and quality of ecosystem service in different units was studied. The results show that the PLAND index of the forest land and the LSI of the brush are significantly associated with the quality of the ecosystem service. This paper mainly studies two associations. 

#### 3.3.1. Analysis of the Association of the Percentage of Patches (PLAND) Index of Forest Land and Quality of Ecosystem Service in Haidian District

**Figure 11:**
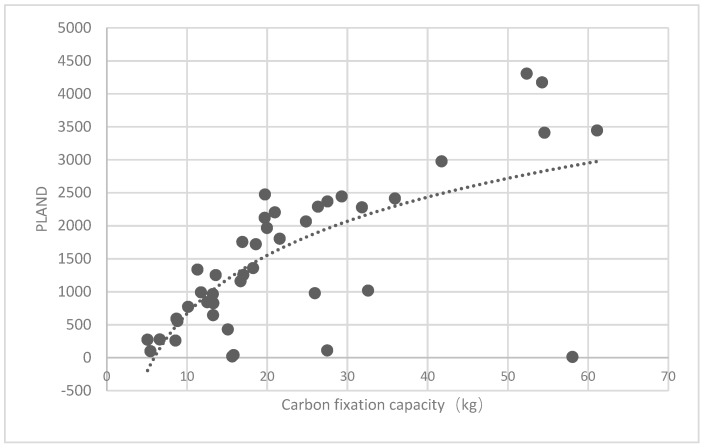
Association between the percentage of patches (PLAND) index for forest land, and carbon fixation capacity.

**Figure 12:**
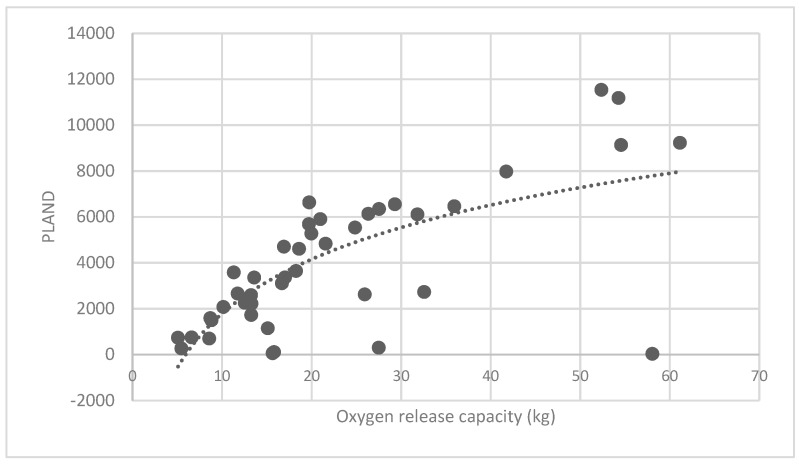
Association between forest land PLAND index and oxygen release capacity.

**Figure 13:**
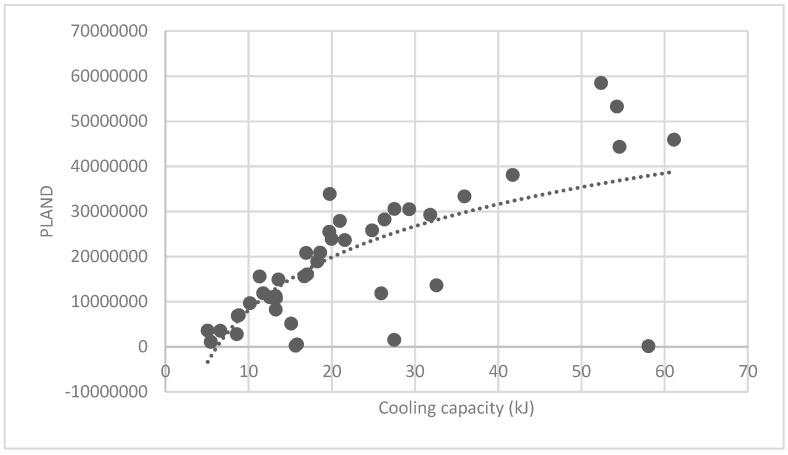
Association between forest land PLAND index and oxygen cooling capacity.

**Figure 14:**
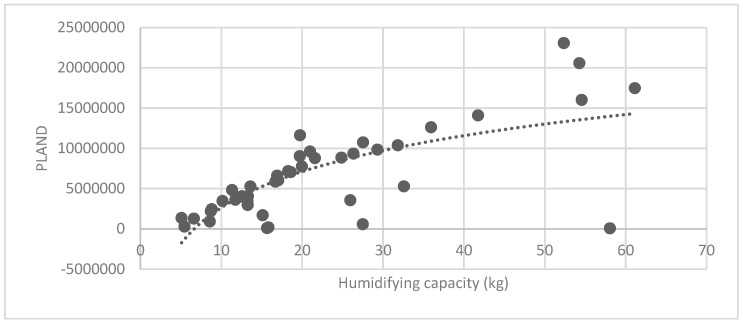
Association between forest land PLAND index and humidifying capacity.

**Figure 15:**
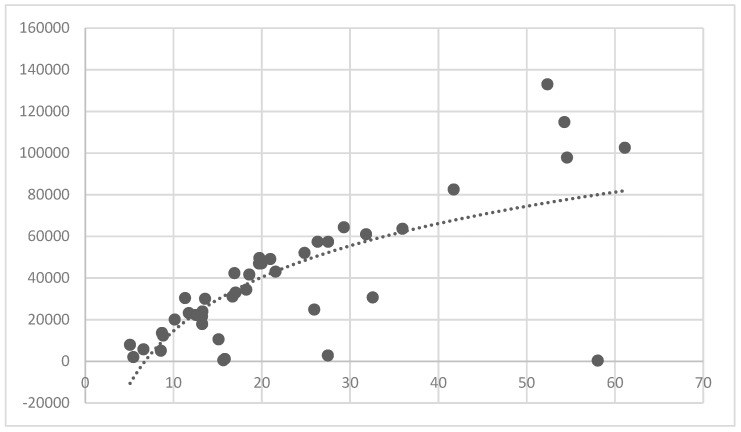
Association between forest land PLAND index and sulfur-dioxide absorption capacity.

**Figure 16:**
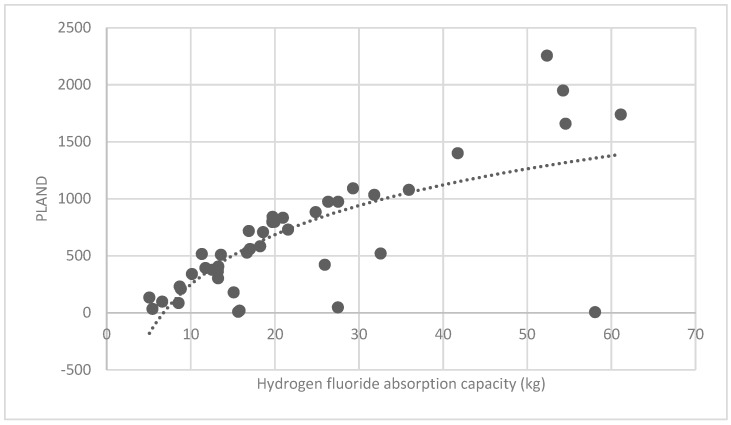
Association between forest land PLAND index and hydrogen-fluoride absorption capacity.

**Figure 17:**
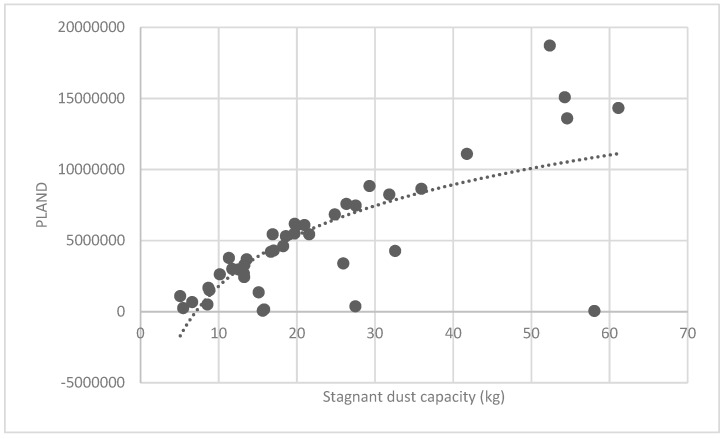
Association between forest land PLAND index and stagnant dust capacity.

**Figure 18:**
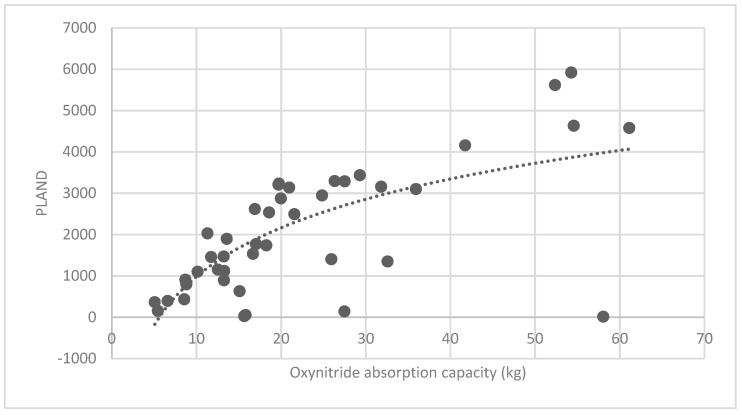
Association between forest land PLAND index and oxynitride absorption capacity.

**Figure 19:**
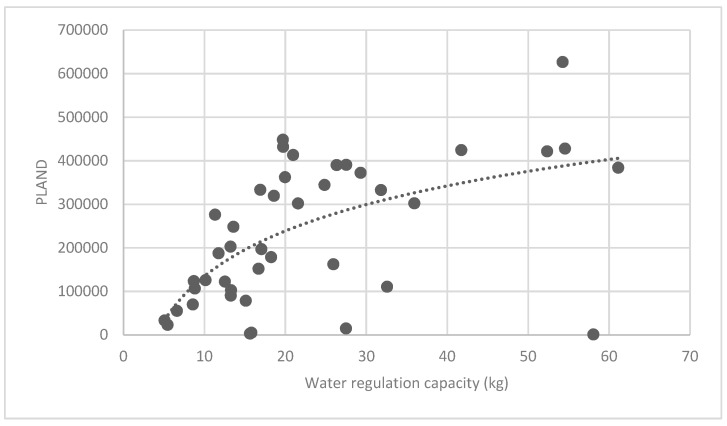
Association between forest land PLAND index and water regulation capacity.

**Figure 20:**
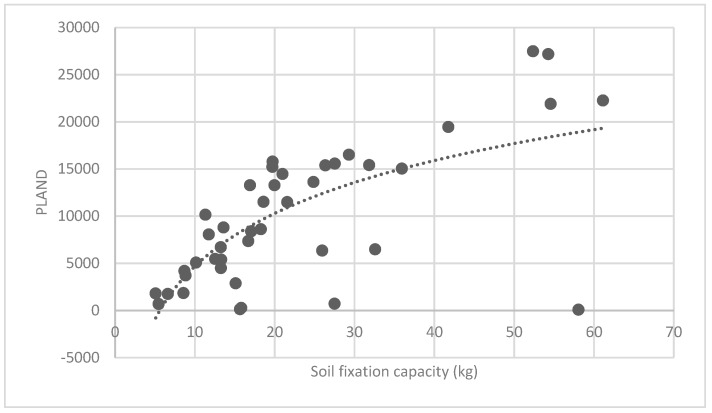
Association between forest land PLAND index and soil fixation capacity.

**Figure 21:**
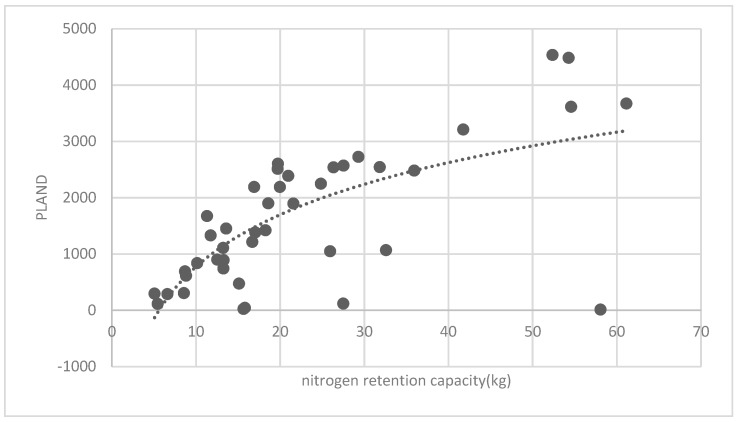
Association between forest land PLAND index and oxynitride absorption capacity.

**Figure 22:**
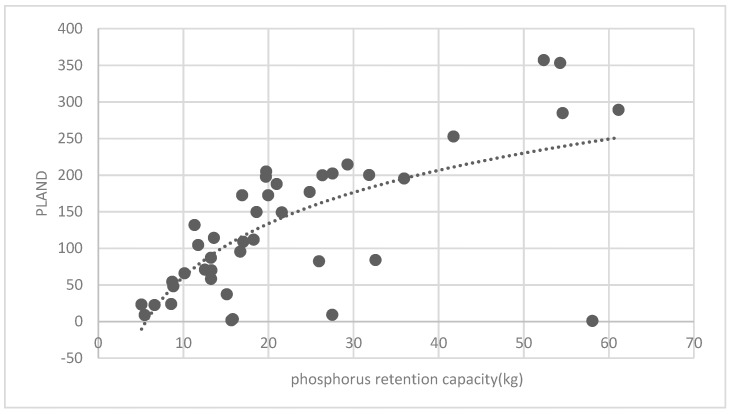
Association between the forest land PLAND index and phosphorous retention capacity.

**Figure 23:**
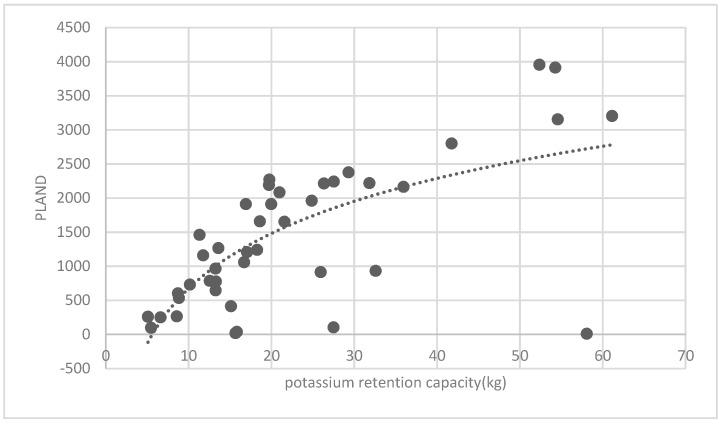
Association between the forest land PLAND index and potassium retention capacity.

**Figure 24:**
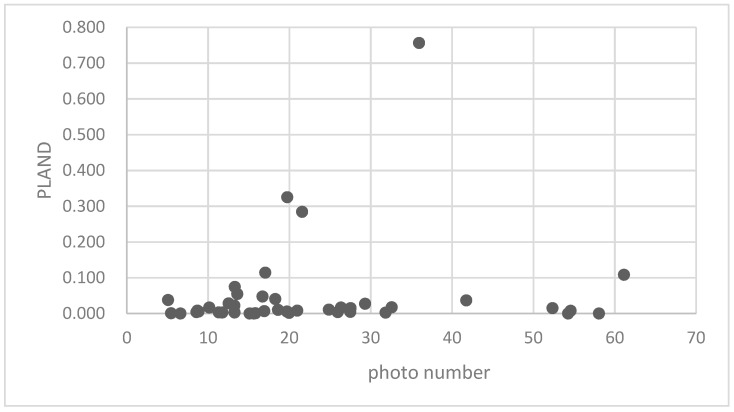
Association between the forest land PLAND index and photo number.

The association between the PLAND index of the forest land and the quality of the ecosystem service in the research units is shown in Figure 11, Figure 12, Figure 13, Figure 14, Figure 15, Figure 16, Figure 17, Figure 18, Figure 19, Figure 20, Figure 21, Figure 22, Figure 23 and Figure 24. The PLAND index has a significant logarithmic relationship with the regulating service and supporting service, however has no significant association with the cultural service (Figure 24). When the PLAND value is between 30 and 40, the regulating services, such as carbon fixation and oxygen release, cooling and humidifying, and air purification, Figure 11, Figure 12, Figure 13, Figure 14, Figure 15, Figure 16, Figure 17, Figure 18 and Figure 19 result. When the PLAND value is more than 40, the growth trend will slow down. The regulation water capacity and soil fixation and fertility retention service (Figure 20, Figure 21, Figure 22 and Figure 23) will result when the PLAND value is between 20 and 30. When the PLAND value is more than 30, the growth will slow down. It is concluded that the quality of the regulating service and supporting service grows quickly when the forest land area is 30% of the landscape area. When the proportion of the forest land is less than 30%, with growth of the proportion of the forest land, the quality of the regulating and supporting service grows quickly. When the proportion is over 30%, the quality growth will slow down.

#### 3.3.2. Analysis of Landscape Shape Index (LSI) of Brush and Quality of Ecosystem Service in Haidian District

**Figure 25:**
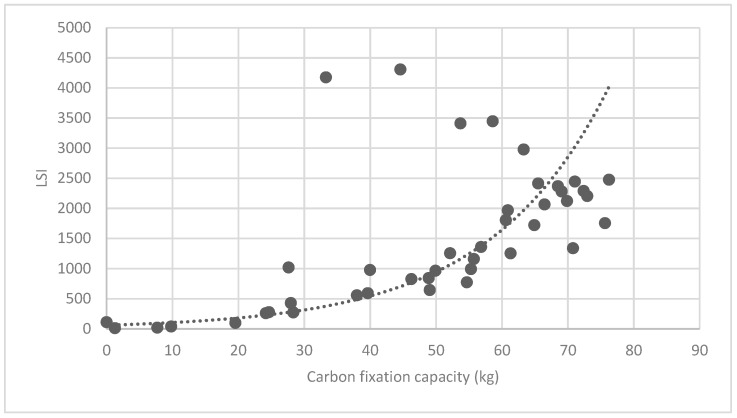
Association between the landscape shape index (LSI) of brush, and carbon fixation capacity.

**Figure 26:**
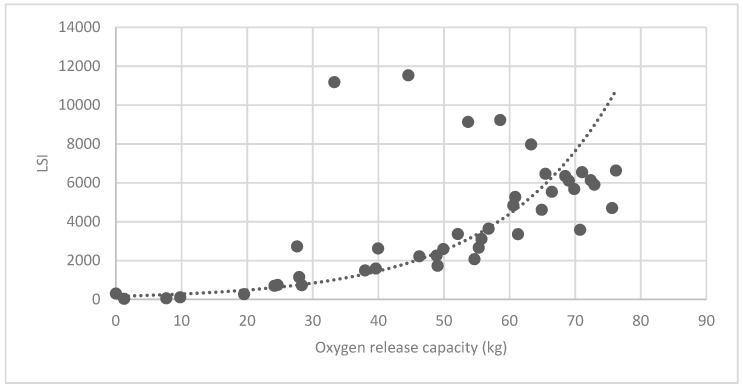
Association between brush LSI and oxygen release capacity.

**Figure 27:**
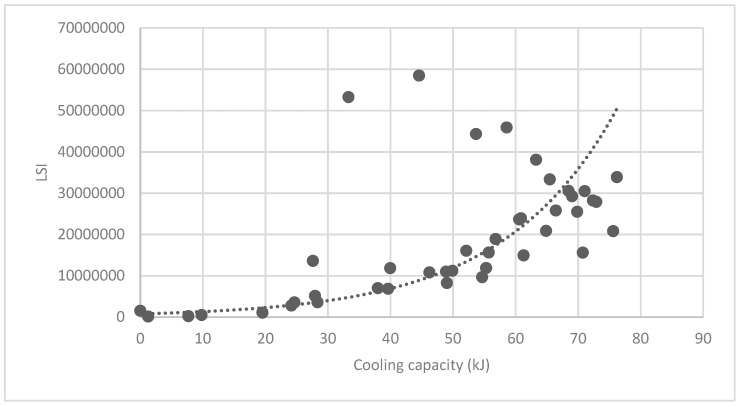
Association between brush LSI and cooling capacity.

**Figure 28:**
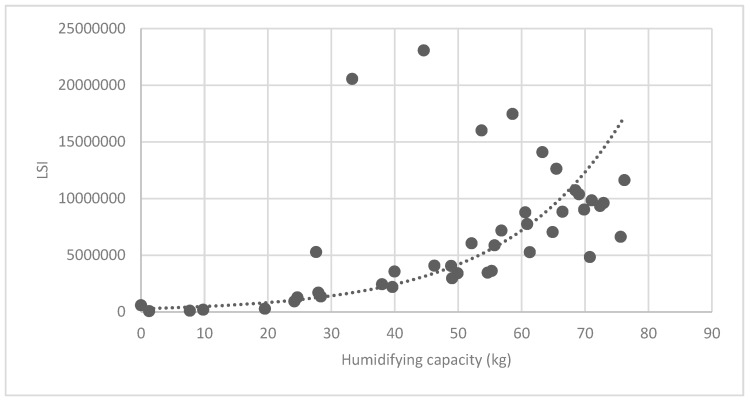
Association between brush LSI and humidifying capacity.

**Figure 29:**
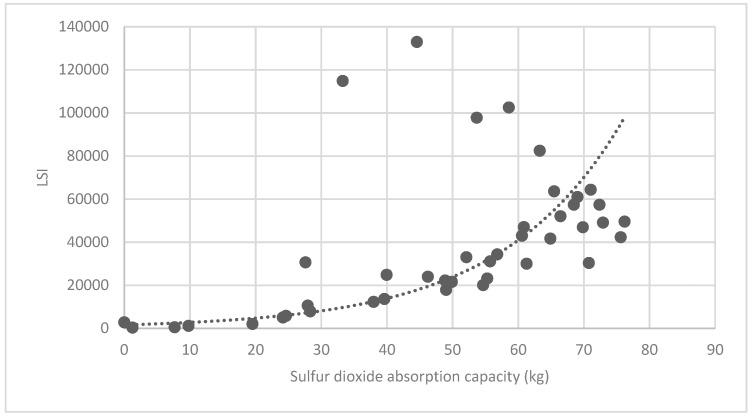
Association between brush LSI and sulfur-dioxide absorption capacity.

**Figure 30:**
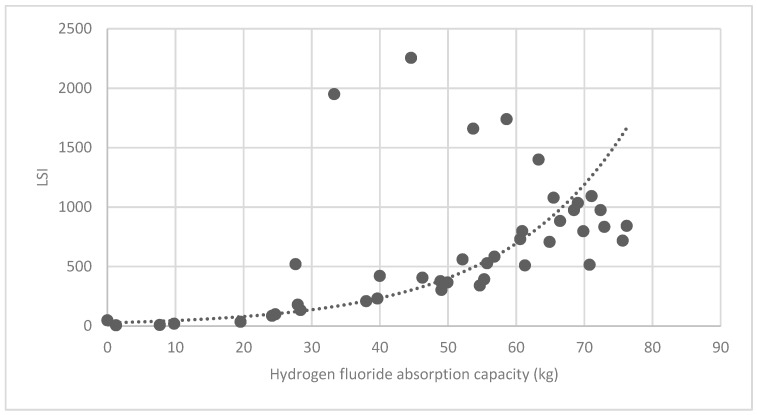
Association between brush LSI and hydrogen-fluoride absorption capacity.

**Figure 31:**
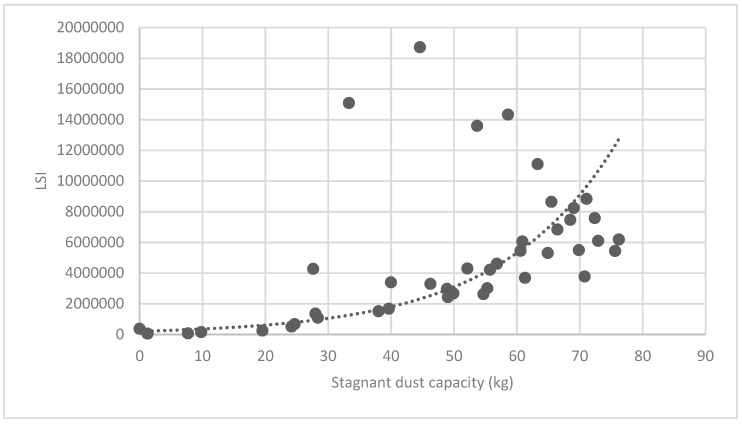
Association between brush LSI and stagnant dust capacity.

**Figure 32:**
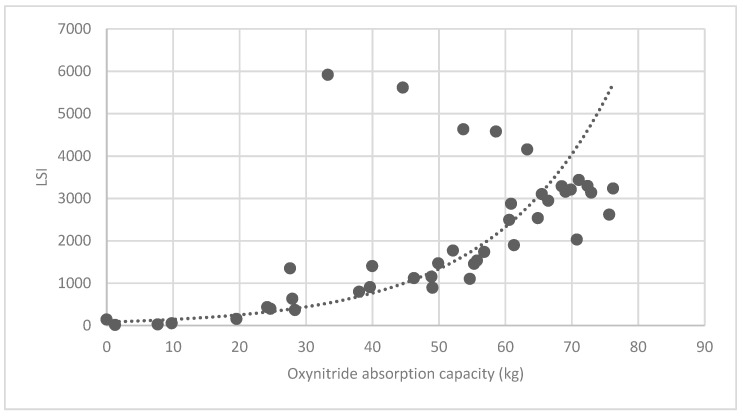
Association between brush LSI and oxynitride absorption capacity.

**Figure 33:**
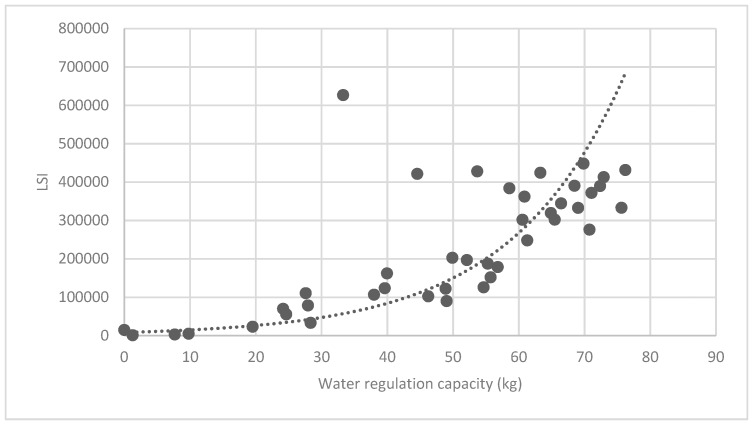
Association between brush LSI and water regulation capacity.

**Figure 34:**
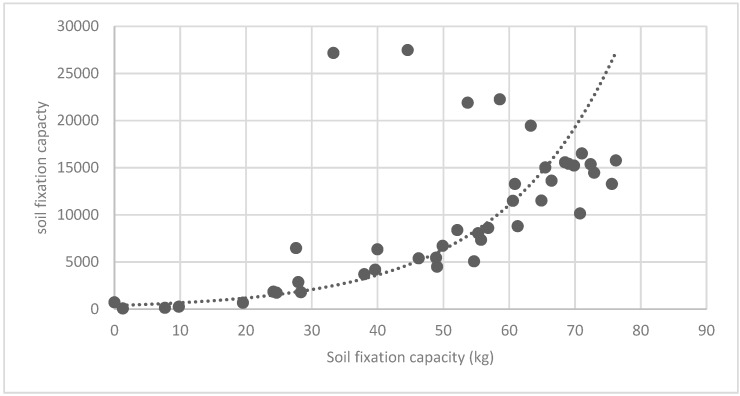
Association between brush LSI and soil fixation capacity.

**Figure 35:**
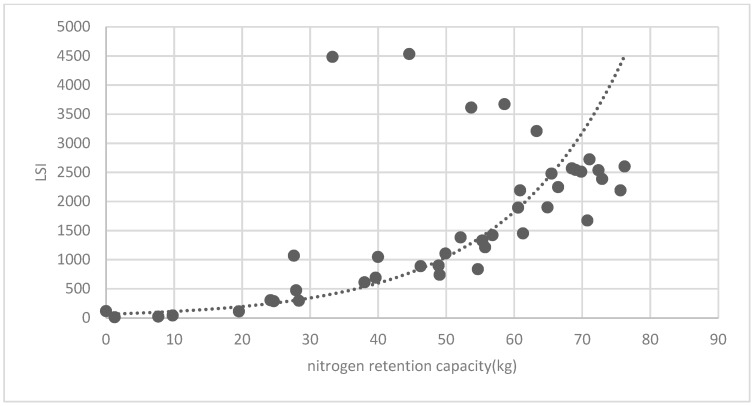
Association between brush LSI and nitrogen retention capacity.

**Figure 36:**
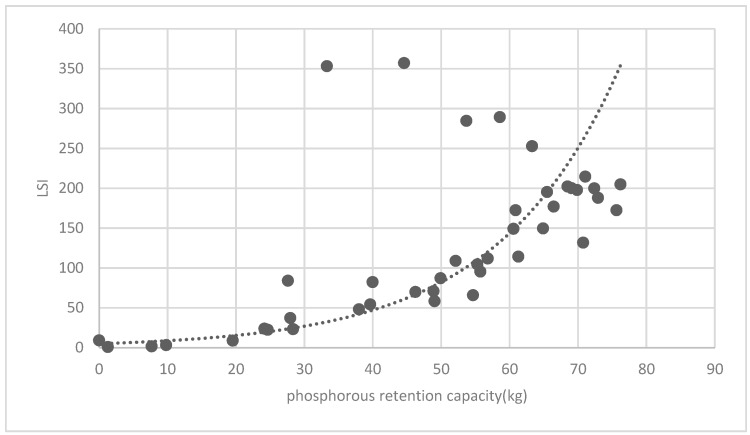
Association between brush LSI and phosphorous retention capacity.

**Figure 37:**
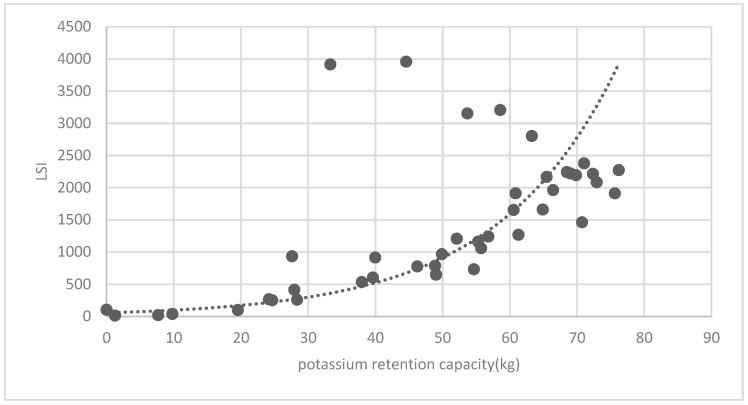
Association between brush LSI and potassium retention capacity.

**Figure 38:**
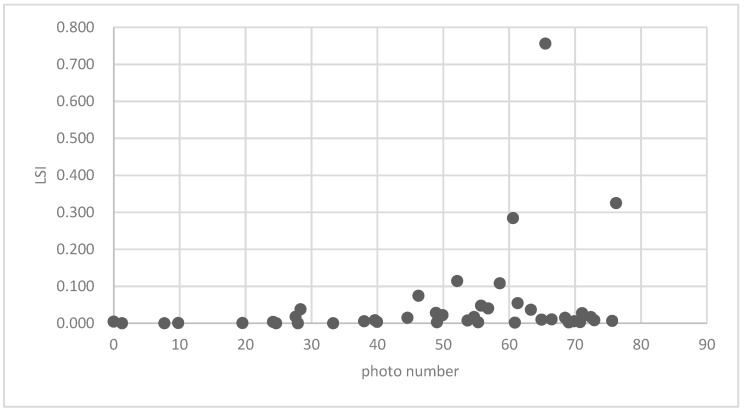
Association between brush LSI and photo number.

Figure 25 show the relationship between the LSI of brush of research units and the quality of ecosystem service in the units. The results show that the LSI of the brush and regulating service (Figure 25, Figure 26, Figure 27, Figure 28, Figure 29, Figure 30, Figure 31, Figure 32 and Figure 33) and supporting service (Figure 34, Figure 35, Figure 36 and Figure 37) show significant exponential growth trends. The cultural service (Figure 38) grows. When the LSI value is 50, the regulating service and supporting service start to grow quickly. It is concluded that the landscape is optimized with 50 as the critical LSI value. The values of units Z16, Z17, Z21, and Z29 in the curve do not satisfy the growth trend. By studying these four units, they are located in the western mountainous areas, and the proportion of the forest land is high (PLAND values are 52.36, 61.14, 54.28, and 54.58, respectively). The quality of the provided ecosystem service is dominant. At this time, it is not associated with the shape index of the brush patches. Therefore, the research results show that the shape index of the brush is exponentially associated with the quality of the ecosystem service, except in the Xishan area with most forest land in Haidian District, and 50 is also the critical value of the LSI.

## 4. Discussion

### 4.1. Association between Landscape Pattern and Ecosystem Service

Research on the ecosystem of urban green spaces can facilitate the protection of the urban ecological system and improve the residential environment. The quantitative evaluation of the ecosystem is the important prerequisite for the protection and improvement of the ecosystem environment. From the view of the ecosystem service, by studying the association between the landscape pattern and service quality, the quantitative ratio of the land coverage type is the important influencing factor for the quality of the ecosystem service. Tang, Shao et al. [65] studied the quality of the ecosystem service in the southern areas of Guizhou Province, China, and proposed that a higher forest land coverage will indicate better ecosystem service condition. Additionally, Zhang et al. [66] studied the karst region in the northwest of Guangxi Province, China, and found that the forest land and brush are the main providers of the ecosystem service, accounting for about 70% of the total service. Furthermore, Cui and Xu [54] studied different ecosystems in Beijing and found that forest land provides the maximal ecosystem service. This research conclusion is consistent with the results of the present study. A high-resolution (1.5 m resolution) remote sensing image is studied in this paper. The data precision is far higher than that in past research. Therefore, the relationships determined in the present quantitative research are more reliable than those of past research. The research results show that the forest land PLAND index has a significant logarithmic correlation with the regulating service and supporting service, with the critical PLAND index value being 30. When this value is less than 30, with growth of the forest land proportion, the quality of the regulating and supporting service will grow quickly. After 30 days, the quality growth will slow down. According to regulations in China’s “Forest Law of the People’s Republic of China”, the forest coverage rate shall reach 30% in China, which matches the present research results. The present research results show that the more complicated landscape shape index will bring a higher quality of ecosystem service under certain conditions. With Ningbo City, China, as one example, Cen concluded that the complexity of the forest land patch will affect the water conservation and soil conservation. The present research results show that the shape index of the brush is exponentially associated with the quality of the ecosystem service, except in the Xishan area with most forest land in Haidian District, and 50 is also the critical value of the LSI value.

### 4.2. Optimization Strategy of Green Space in Haidian District

According to the present research results, from the view of the ecosystem service, this paper proposes an optimization strategy for the green space in Haidian District, Beijing City. The ecosystem service functions are better in the western mountainous areas with higher forest coverage. Therefore, these areas should be protected and an ecological red line should be drawn to strictly control city expansion. The regulating and supporting services are better in the northern plain area. Under the prerequisite of protecting the ecosystem, the ecosystem service is further enhanced by improving the patch shape index. The cultural service is better and the regulating and supporting services are worse in the southeast of the city. By referring to the research results, the forest land proportion should be added in the existing green space to improve the ecosystem service capability of the urban area and enhance the service capability of the urban ecosystem. 

## 5. Conclusions 

Using a high-resolution (1.5 m resolution) remote sensing image from the GF-2 satellite as the foundation, the quality of the ecosystem service in Haidian District, Beijing, China, was evaluated, and the research units were divided to study the association between the landscape pattern and ecosystem service. The following conclusions are drawn:

(1) The research results show that forest land is the main provider of the ecosystem service in Haidian District, while construction land only provides cultural service. For the total quality of the unit area of the ecosystem service, the quality of units Z10, Z11, Z16, Z17, Z18, Z19, Z21, Z22, Z29, and Z31 is higher than the quality of unit Z30 approximates the whole average in Haidian District;
(2) On the whole, the ecosystem service spaces of Haidian District are divided into the western mountainous area, the northern plain, and the southwestern urban area, and decrease from the west to the east. These are roughly matched with the spatial distribution of the forest land. The regulating service and supporting service are maximum in the western mountainous area, followed by the northern plain. The southwestern urban area has the minimal regulating service and supporting service. This is consistent with the distribution of the urban green space. The cultural service capabilities in the research unit where there are important historical cultural resources in Haidian District—namely, the “Three Hills and Five Parks”—are far stronger than those of other areas. Therefore, the cultural service is closely associated with the historical resource points;
(3) The PLAND index of the forest land has a significant logarithmic relationship with the regulating service and supporting service in the analysis on association between the landscape pattern and ecosystem service quality. The critical PLAND index value is 30. When the PLAND value is smaller than 30, with growth of the proportion of the forest land, the quality of the regulating and supporting service will grow quickly. When the value is over 30, the quality growth will slow down. Additionally, the research results show that the shape index of the brush is exponentially associated with the quality of the ecosystem service, except in the Xishan area with most forest land in Haidian District (the quality of the ecosystem service of the forest land is dominant, and at this time it is not associated with the shape index of the brush spot). The critical LSI value is 50;
(4) Finally, this paper proposes an area optimization strategy for green space in Haidian District, Beijing City, from the view of the ecosystem system service. The Xishan area is classified as the ecosystem red line to control city expansion. The regulating and supporting services can be enhanced in the northern flat area by improving the patch shape index. The ecosystem service capabilities can be improved by adding forest land in the existing green space for the southeastern urban areas.

This paper studies the quality of the ecosystem service, however, does not cover research on biological diversity conservation. This will be studied later. The research results show that the distribution of the regulating and supporting service is roughly the reverse of that of the cultural service. Therefore, it is concluded that the ecosystem services are coordinated and balanced with each other, and the relation mechanism shall be further studied to provide theoretical support for urban construction decisions.

## Figures and Tables

**Table 1:** Land coverage type determination mark of picture.

Type of Land Coverage	Determination Mark	Picture
Forest land	Located in mountainous areas. The image is deep red, grainy, and has even texture.	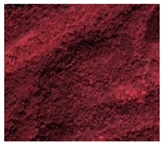
Brush	Mainly located at the foot of hills and inside the city. This image includes deep red and shiny red and is grainy.	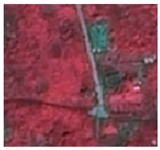
Grasslands	Its area is small inside the city. The image is shiny red.	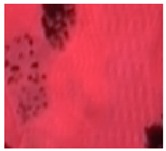
Construction land	Distributed in the constructed urban area. The image is cyan and has distinctive geometric features.	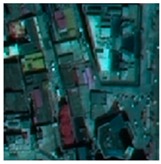
Water area	The image is deep cyan and has a distinctive boundary and fine texture.	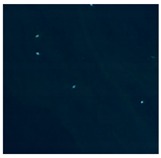
Other lands	Mainly located outside the city. The image is light gray and white.	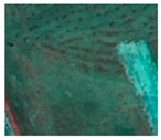

**Table 2:** Quality of ecosystem services of different land coverage types.

Land Use and Land Cover Type	Regulating Service									Supporting Service				Cultural Service
	Carbon Fixation And Oxygen Release		Cooling and Humidifying		Air Purification				Water Conservation	Soil Fixation Capacity	Fertility Conservation Capacity			Landscape Quality
	Carbon Fixation Capacity(Kg)	Oxygen Release Capacity (Kg)	Cooling Capacity (Kj)	Humidifying Capacity (Kg)	Sulfur-Dioxide Abstraction Capacity (Kg)	Oxynitride Absorption Capacity (Kg)	Hydrogen-Fluoride Absorption Capacity (Kg)	Dust Capacity (Kg)	Regulation Water Capacity (Kg)	Solid Fixation Capacity (Kg)	Nitrogen Capacity (Kg)	Phosphorous Retention Capacity (Kg)	Kalium Retention Capacity (Kg)	Photo Number
Non-utilized land	0.00	0.00	0.00	0.00	0.00	0.00	0.00	0.00	0.00	28,579.58	4715.63	371.53	4115.46	177
Brush	19,180.28	51, 348.66	200,744,800.46	18,981,221.98	374,230.23	29,521.19	6347.05	53,285,740.10	4,317,473.40	125,858.65	20,766.68	1636.16	18,123.65	297
Construction	0.00	0.00	0.00	0.00	0.00	0.00	0.00	0.00	0.00	0.00	0.00	0.00	0.00	847
Forest land	56,440.90	151,101.28	785,246,088.49	320,154,576.94	1,815,492.32	71,602.93	30,789.26	258,605,919.41	4,537,835.76	347,632.23	57,359.32	4519.22	50,059.04	1007
Grassland	14,346.55	38,408.01	13,111,226.57	53,592,790.68	178,667.05	28,188.33	3053.74	1,310,757.48	5,689,110.28	112,659.37	18,588.80	1464.57	16,222.95	459
Water area	2371.38	6348.56	61,303,263.82	24,990,252.41	0.00	0.00	0.00	0.00	0.00	0.00	0.00	0.00	0.00	576

**Table 3:** Quality of ecosystem services of unit areas of research units.

UNIT	Regulating Service									Supporting Service				Cultural Services
	Carbon Fixation and Oxygen Release		Cooling and Humidifying		Air Purification				Water Conservation	Soil Capacity	Fertility Retention Capacity			Landscape Quality
	Carbon Fixation Capacity (kg)	Oxygen Release Capacity (kg)	Cooling Capacity (kJ)	Humidifying Capacity (kg)	Sulfur Dioxide Absorption Capacity (kg)	Oxynitride Absorption Capacity (kg)	Hydrogen Fluoride Absorption Capacity(kg)	Stagnant Dust capacity (kg)	Regulation Water Capacity (kg)	Soil Fixation Capacity (kg)	Nitrogen Retention Capacity (kg)	Phosphorous Retention Capacity (kg)	Potassium Retention Capacity (kg)	Photo Number
Z01	98.86	264.66	1,089,370.95	278,651.71	2029.42	155.75	34.46	246,952.66	23,114.25	680.81	112.33	8.85	98.04	0.00
Z02	644.02	1724.16	824,5341.47	2,965,043.41	17,843.74	893.99	302.73	2,430,765.79	90,016.27	4488.63	740.62	58.35	646.36	0.00
Z03	1358.69	3637.43	18,922,395.65	7,166,506.47	34,361.95	1738.63	583.04	4,603,731.79	178,531.66	8609.15	1420.51	111.92	1239.72	0.04
Z04	110.22	295.09	1,536,790.67	584,776.61	2776.80	141.09	47.12	370,682.43	14,584.04	711.59	117.41	9.25	102.47	0.00
Z05	992.03	2655.82	11,897,912.59	3,614,960.46	23,155.86	1457.70	393.00	3,007,780.79	187,409.15	8046.84	1327.73	104.61	1158.74	0.00
Z06	1754.57	4697.27	20,826,206.51	6,617,409.13	42,285.39	2,618.30	717.71	5,439,770.40	333,108.04	13,271.60	2189.81	172.53	1911.11	0.01
Z07	1159.69	3104.67	15,638,070.60	5,872,025.54	31,094.95	1,533.76	527.56	4,213,242.78	151,712.59	7350.16	1212.78	95.55	1058.42	0.05
Z08	272.68	730.01	3,598,599.99	1,349,254.21	7883.77	366.89	133.73	1,090,693.73	33,058.13	1791.80	295.65	23.29	258.02	0.04
Z09	1017.61	2724.30	13,620,410.84	5,280,237.49	30,637.44	1350.17	519.67	4,275,282.07	110,540.70	6471.18	1067.74	84.13	931.85	0.02
Z10	2444.64	6544.69	30,499,332.87	9,836,865.91	64,361.80	3435.97	1091.86	8,838,893.85	371,932.04	16,506.10	2723.51	214.58	2376.88	0.03
Z11	2475.51	6627.34	33,885,723.79	11,622,445.09	49,558.04	3234.71	841.34	6,184,853.58	431,598.88	15,769.13	2601.91	205.00	2270.75	0.33
Z12	1254.29	3357.93	16,053,160.64	6,044,347.02	32,990.58	1770.74	559.89	4,297,271.60	196,687.32	8380.05	1382.71	108.94	1206.73	0.11
Z13	825.73	2210.62	10,820,779.16	4,072,016.48	23,927.26	1121.23	405.90	3,296,572.88	102,326.90	5388.33	889.07	70.05	775.92	0.07
Z14	39.03	104.48	508,925.66	190,532.11	1122.65	53.28	19.04	154,174.32	4964.20	255.04	42.08	3.32	36.73	0.00
Z15	11.04	29.54	153,276.89	62,293.31	354.00	14.02	6.00	50,406.92	899.46	68.01	11.22	0.88	9.79	0.00
Z16	4306.32	11,528.72	58,485,294.22	23,067,885.31	132,947.67	5616.88	2254.90	18,719,387.88	421,461.26	27,470.03	4532.56	357.11	3955.68	0.01
Z17	3445.06	9222.98	45,908,339.00	17,463,742.11	102,528.69	4580.14	1739.08	14,327,857.48	383,735.16	22,249.93	3671.24	289.25	3203.99	0.11
Z18	2413.15	6460.37	33,359,198.32	12,620,545.62	63,591.12	3102.81	1078.87	8,641,584.33	302,093.20	15,027.10	2479.47	195.35	2163.90	0.76
Z19	1804.30	4830.40	23,652,766.43	8,782,159.02	43,000.84	2495.35	729.93	5,443,177.37	301,894.22	11,478.61	1893.97	149.22	1652.92	0.28
Z20	841.79	2253.59	11,003,446.57	4,048,156.36	22,226.48	1152.85	377.14	2,967,955.60	122,113.09	5451.04	899.42	70.86	784.95	0.03
Z21	4174.34	11,175.37	53,256,159.48	20,560,698.51	114,850.11	5919.35	1949.03	15,081,816.33	626,560.01	27,167.69	4482.67	353.18	3912.15	0.00
Z22	2976.66	7968.99	38,109,493.67	14,086,158.38	82,469.26	4158.21	1399.25	11,102,007.08	424,272.57	19,447.45	3208.83	252.82	2800.43	0.04
Z23	2280.10	6104.20	29,261,291.47	10,377,309.17	60,981.91	3159.30	1034.65	8,241,275.62	332,561.04	15,404.64	2541.76	200.26	2218.27	0.00
Z24	2065.43	5529.48	25,808,837.66	8,838,491.05	52,043.29	2946.78	883.18	6,843,307.78	344,357.89	13,618.26	2247.01	177.04	1961.03	0.01
Z25	1720.27	4605.44	20,862,880.06	7,039,270.69	41,665.70	2537.09	707.24	5,307,363.69	319,282.49	11,508.88	1898.96	149.62	1657.28	0.01
Z26	1252.72	3353.72	14,941,163.10	5,259,749.86	30,006.27	1896.38	509.47	3,683,063.26	248,102.26	8790.34	1450.41	114.27	1265.81	0.05
Z27	772.21	2067.34	9,685,283.47	3,462,063.18	20,035.40	1103.73	340.01	2,626,304.53	125,670.01	5070.79	836.68	65.92	730.19	0.02
Z28	277.14	741.94	3,553,896.42	1,274,534.50	5756.73	395.03	97.77	674,924.88	55,272.85	1738.43	286.84	22.60	250.33	0.00
Z29	3409.75	9128.46	44,331,653.58	16,005,657.53	97,783.56	4633.20	1658.67	13,595,988.24	427,829.40	21,897.90	3613.15	284.67	3153.30	0.01
Z30	2,289.91	6130.46	28,221,265.28	9,338,148.84	57,401.49	3296.12	974.08	7,578,555.21	389,831.71	15,374.35	2536.77	199.87	2213.91	0.02
Z31	2368.65	6341.25	30,554,575.51	10,733,548.30	57,400.27	3290.20	974.17	7, 466, 149.98	390,339.76	15,562.46	2567.81	202.31	2240.99	0.02
Z32	2120.60	5677.19	25,508,689.05	9,026,399.78	46,906.70	3209.01	796.67	5,497,912.02	448,152.65	15,221.63	2511.57	197.88	2191.91	0.01
Z33	1336.63	3578.38	15,598,446.89	4,830,956.52	30,344.56	2031.25	515.16	3,779,051.51	275,999.23	10,140.76	1673.23	131.83	1460.27	0.00
Z34	259.69	695.23	2,799,377.97	929,508.65	5054.12	433.65	85.92	509,150.49	69,797.08	1841.38	303.83	23.94	265.16	0.00
Z35	19.10	51.13	242,718.12	92,488.41	522.23	27.13	8.86	68,662.22	2896.77	132.29	21.83	1.72	19.05	0.00
Z36	977.37	2616.57	11,846,800.72	3,551,967.99	24,814.14	1405.03	420.98	3,390,078.31	162,088.86	6340.20	1046.13	82.42	912.99	0.00
Z37	965.42	2584.59	11,217,703.68	3,397,937.30	21,547.69	1470.35	365.82	2,675,715.17	202,521.02	6703.00	1106.00	87.14	965.23	0.02
Z38	1967.22	5266.56	23,918,451.96	7,747,075.91	46,978.34	2875.38	797.35	6,061,153.49	362,107.99	13,273.25	2190.09	172.55	1911.35	0.00
Z39	2203.63	5899.48	27,902,641.09	9,603,191.19	49,100.05	3138.55	833.59	6,096,387.62	412,946.76	14,460.32	2385.95	187.98	2082.29	0.01
Z40	556.22	1489.08	7,005,562.57	2,433,737.50	12,312.70	798.94	209.06	1,509,276.71	106,601.53	3705.51	611.41	48.17	533.59	0.01
Z41	427.42	1144.28	5,136,519.60	1,697,036.12	10,530.48	632.83	178.73	1,358,032.37	78,498.06	2865.72	472.84	37.25	412.66	0.00
Z42	592.07	1585.06	6,872,312.66	2,192,461.65	13,598.89	907.99	230.89	1,675,970.81	123,450.50	4181.65	689.97	54.36	602.16	0.01
AVG	2144.59	5741.41	27,375,619.42	9,701,586.37	55,006.22	3003.30	933.42	7,274,175.82	337,796.45	14,277.20	2355.74	185.60	2055.92	0.08

**Table 4:** Landscape pattern indices of research units.

UNIT	TYPE	PLAND ^1^	LSI ^2^	IJI ^3^
Z01	Other land	0.66	5.29	71.81
	Brush	14.72	19.54	55.16
	Construction land	69.64	13.26	67.58
	Forest land	5.46	11.91	72.18
	Grasslands	9.33	15.17	61.22
	Water area	0.19	2.26	51.57
Z02	Other land	2.23	20.00	71.95
	Brush	4.28	49.04	62.9
	Construction land	77.21	28.18	66.27
	Forest land	13.26	47.24	63.00
	Grasslands	2.96	37.35	69.16
	Water area	0.05	4.51	68.58
Z03	Other land	2.57	26.97	63.70
	Brush	4.12	56.82	72.57
	Construction land	66.34	33.59	61.25
	Forest land	18.26	58.9	70.57
	Grasslands	5.43	50.63	73.34
	Water area	3.28	6.30	71.41
Z04	Other land	13.75	3.86	22.24
	Construction land	58.75	2.21	65.34
	Forest land	27.50	14.11	57.18
Z05	Other land	9.20	32.51	62.00
	Brush	12.68	55.30	70.40
	Construction land	58.28	37.07	75.19
	Forest land	11.75	39.18	78.09
	Grasslands	7.40	35.58	72.89
	Water area	0.68	4.41	63.95
Z06	Other land	7.00	33.61	83.14
	Brush	14.07	75.63	80.46
	Construction land	51.07	37.20	78.86
	Forest land	16.91	61.30	81.70
	Grasslands	10.88	57.06	78.14
	Water area	0.08	4.52	57.40
Z07	Other land	1.11	19.08	62.11
	Brush	3.78	55.73	66.61
	Construction land	72.90	28.59	51.11
	Forest land	16.70	62.65	60.50
	Grasslands	4.03	45.95	70.93
	Water area	1.49	8.39	69.60
Z08	Other land	0.47	8.92	81.27
	Brush	1.02	28.35	62.46
	Construction land	92.67	15.67	56.01
	Forest land	5.08	34.65	48.47
	Grasslands	0.70	20.23	66.29
	Water area	0.05	5.78	58.57
Z09	Other land	0.63	12.90	70.82
	Brush	3.73	27.61	64.29
	Construction land	60.03	19.32	75.67
	Forest land	32.58	12.67	67.81
	Grasslands	3.02	22.10	46.53
	Water area	0	1.22	37.76
Z10	Other land	3.69	35.47	64.50
	Brush	19.51	71.07	65.47
	Construction land	41.04	41.86	62.22
	Forest land	29.31	39.20	68.13
	Grasslands	6.13	39.65	63.47
	Water area	0.34	5.53	66.49
Z11	Other land	6.78	42.92	76.93
	Brush	17.16	76.23	73.49
	Construction land	29.15	46.22	74.22
	Forest land	19.74	59.23	77.81
	Grasslands	17.00	61.61	75.86
	Water area	10.16	7.61	64.43
Z12	Other land	1.37	17.36	68.83
	Brush	3.76	52.12	75.55
	Construction land	69.71	27.17	56.17
	Forest land	17.04	60.77	66.76
	Grasslands	7.50	38.24	74.70
	Water area	0.61	7.24	71.63
Z13	Other land	0.64	13.10	54.00
	Brush	2.55	46.25	61.54
	Construction land	81.42	22.31	46.99
	Forest land	13.30	51.07	48.23
	Grasslands	2.09	31.59	62.93
	Water area	0.01	3.53	44.49
Z14	Other land	0.73	2.83	77.26
	Brush	3.26	9.80	65.93
	Construction land	77.45	6.17	78.75
	Forest land	15.82	8.90	69.48
	Grasslands	2.74	9.02	72.13
Z15	Brush	0.39	1.27	0
	Construction land	41.48	3.78	49.97
	Forest land	58.07	1.46	70.58
	Grasslands	0.06	1.00	0
Z16	Other land	1.74	25.37	66.3
	Brush	3.82	44.58	66.83
	Construction land	39.39	30.06	72.36
	Forest land	52.36	15.65	75.41
	Grasslands	2.67	27.77	43.62
	Water area	0.01	1.84	67.15
Z17	Other land	2.80	23.24	73.89
	Brush	9.82	58.59	70.65
	Construction land	20.68	28.41	64.97
	Forest land	61.14	26.42	79.77
	Grasslands	5.38	36.42	56.66
	Water area	0.19	3.00	72.52
Z18	Other land	2.86	28.31	80.39
	Brush	7.67	65.49	77.71
	Construction land	40.62	34.19	71.91
	Forest land	35.95	41.70	83.50
	Grasslands	8.10	47.45	82.63
	Water area	4.80	8.28	77.04
Z19	Other land	1.26	19.18	75.62
	Brush	6.75	60.58	76.18
	Construction land	53.82	31.35	68.00
	Forest land	21.57	60.89	76.07
	Grasslands	13.27	49.66	75.18
	Water area	3.33	9.19	80.47
Z20	Other land	0.80	9.71	64.46
	Brush	3.61	48.89	68.00
	Construction land	78.21	22.55	61.80
	Forest land	12.54	45.40	64.00
	Grasslands	4.05	36.16	68.03
	Water area	0.79	8.38	80.11
Z21	Other land	0.17	9.79	81.42
	Brush	7.56	33.29	55.43
	Construction land	17.15	20.68	73.75
	Forest land	54.28	21.00	43.87
	Grasslands	20.85	31.27	38.91
	Water area	0	1.10	56.98
Z22	Other land	1.25	25.77	75.74
	Brush	10.46	63.30	66.81
	Construction land	34.89	27.07	64.65
	Forest land	41.75	32.59	68.48
	Grasslands	11.54	43.28	58.92
	Water area	0.10	4.05	70.47
Z23	Other land	4.14	32.12	79.3
	Brush	12.16	69.05	76.88
	Construction land	42.18	33.47	80.30
	Forest land	31.83	42.12	83.52
	Grasslands	8.65	42.55	76.83
	Water area	1.03	9.87	79.97
Z24	Other land	1.53	25.38	85.72
	Brush	13.19	66.45	74.40
	Construction land	48.25	30.65	74.54
	Forest land	24.85	41.59	82.58
	Grasslands	11.09	47.94	75.34
	Water area	1.09	10.56	77.30
Z25	Other land	1.00	17.27	86.23
	Brush	11.48	64.90	71.43
	Construction land	56.13	31.37	71.38
	Forest land	18.60	41.01	74.63
	Grasslands	12.21	40.58	73.43
	Water area	0.59	8.51	85.9
Z26	Other land	1.80	17.71	81.11
	Brush	6.52	61.30	65.23
	Construction land	66.46	37.10	64.17
	Forest land	13.59	62.54	59.55
	Grasslands	11.50	45.05	67.74
	Water area	0.14	3.71	65.32
Z27	Other land	0.34	6.30	75.93
	Brush	3.81	54.67	59.47
	Construction land	81.03	26.96	59.26
	Forest land	10.15	55.17	50.45
	Grasslands	4.43	34.36	65.64
	Water area	0.23	4.98	72.53
Z28	Other land	0.19	7.16	86.48
	Brush	3.77	24.65	72.16
	Construction land	79.70	13.84	76.01
	Forest land	6.61	20.91	75.89
	Grasslands	7.79	17.07	74.05
	Water area	1.93	7.00	71.89
Z29	Other land	1.36	20.30	67.91
	Brush	16.19	53.69	60.01
	Construction land	21.40	23.51	63.30
	Forest land	54.58	27.55	53.28
	Grasslands	6.46	42.68	47.44
	Water area	0.02	2.33	80.08
Z30	Other land	2.51	25.43	84.94
	Brush	16.96	72.39	75.23
	Construction land	41.78	35.48	71.48
	Forest land	26.34	45.49	80.24
	Grasslands	11.61	47.93	73.9
	Water area	0.80	9.14	75.18
Z31	Other land	3.41	28.62	71.28
	Brush	13.50	68.49	77.8
	Construction land	38.33	31.50	81.03
	Forest land	27.53	43.89	82.87
	Grasslands	13.81	49.84	76.46
	Water area	3.41	12.14	75.42
Z32	Other land	5.30	34.84	72.40
	Brush	10.76	69.86	76.61
	Construction land	39.31	37.89	86.12
	Forest land	19.70	39.12	83.56
	Grasslands	23.02	41.24	81.32
	Water area	1.91	8.47	84.55
Z33	Other land	5.46	25.44	58.27
	Brush	11.97	70.77	63.58
	Construction land	60.34	39.33	73.77
	Forest land	11.32	46.34	72.04
	Grasslands	10.56	44.27	66.33
	Water area	0.35	6.61	68.59
Z34	Other land	0.16	6.52	67.95
	Brush	9.67	24.17	57.96
	Construction land	57.61	20.82	67.87
	Forest land	8.57	18.97	63.05
	Grasslands	23.93	21.21	62.07
	Water area	0.06	1.21	28.38
Z35	Other land	1.97	2.57	49.8
	Brush	2.75	7.69	75.92
	Construction land	73.61	5.64	76.48
	Forest land	15.65	8.01	73.66
	Grasslands	6.01	6.57	75.51
Z36	Other land	0.20	3.70	66.07
	Brush	24.45	39.98	62.19
	Construction land	42.54	26.83	40.74
	Forest land	25.95	24.15	45.84
	Grasslands	6.86	28.59	41.42
	Water area	0	1.17	59.21
Z37	Other land	2.17	20.84	76.08
	Brush	15.93	49.91	66.16
	Construction land	54.85	30.59	69.00
	Forest land	13.24	35.47	75.19
	Grasslands	13.26	29.74	65.43
	Water area	0.56	7.03	64.31
Z38	Other land	2.20	27.43	64.59
	Brush	15.70	60.88	68.76
	Construction land	48.82	32.75	70.69
	Forest land	19.98	36.58	68.70
	Grasslands	12.25	40.38	66.55
	Water area	1.05	6.22	51.72
Z39	Other land	2.69	32.62	66.24
	Brush	13.87	72.93	69.52
	Construction land	41.05	35.94	76.67
	Forest land	20.97	46.04	72.48
	Grasslands	17.46	49.18	68.9
	Water area	3.96	9.21	76.52
Z40	Other land	1.39	12.51	69.89
	Brush	5.56	37.99	72.20
	Construction land	74.58	19.45	80.23
	Forest land	8.83	22.16	77.36
	Grasslands	8.01	25.46	72.58
	Water area	1.63	6.32	89.73
Z41	Other land	0.66	12.48	74.28
	Brush	10.04	27.95	69.13
	Construction land	65.21	14.03	74.91
	Forest land	15.11	16.46	64.00
	Grasslands	8.91	20.56	63.07
	Water area	0.07	2.63	66.01
Z42	Other land	1.47	17.66	72.36
	Brush	7.85	39.63	71.74
	Construction land	73.56	19.24	75.59
	Forest land	8.70	24.93	72.80
	Grasslands	8.36	26.4	67.77
	Water area	0.05	3.81	62.56

^1^ The PLAND index indicates the percentage of the patch in the landscape and describes the quality characteristics of the research area. See document Fragstats Help [64] for more details of the algorithm. ^2^ The interspersion and juxtaposition index (IJI)index is used to express the general distribution of the landscape. See document Fragstats Help [64] for more details of the algorithm. ^3^ The Landscape shape index (LSI) index measures the perimeter-to-area ratio for the landscape as a whole. See document Fragstats Help [64] for more details of the algorithm.

## References

[1] Millennium Ecosystem Assessment (2003). Ecosystem and Human Well-Being: A Framework for Assessment.

[2] Che S., Wang H. (2001). A summary of study on urban green space. J. Shanghai Jiaotong Univ. (Agric. Sci.).

[3] Mao Q.Z., Luo S.H., Ma K.M., Wu J.G., Tang R.L., Zhang Y.X., Le B., Zhang T. (2012). Research advances in ecological assessment of urban green space. Acta Ecol. Sin..

[4] Li F., Wang R. (2004). Research advance in ecosystem service of urban green space. Chin. J. Appl. Ecol..

[5] Liu J., Tian Y., Zhang L. (2010). Study on the Relation of Urban Green Space Area and Ecological Response in Beijing City, China. Conference on Environmental Pollution and Public Health.

[6] Simonich S.L., Hites R.A. (1994). Importance of vegetation in removing polycyclic aromatic hydrocarbons from the atmosphere. Nature.

[7] Beckett K.P., Freersmith P.H., Taylor G. (1998). Urban woodlands: Their role in reducing the effects of particulate pollution. Environ. Pollut..

[8] Bealey W.J., Mcdonald A.G., Nemitz E., Donovan R., Dragosits U., Duffy T.R., Fowler D. (2007). Estimating the reduction of urban PM10 concentrations by trees within an environmental information system for planners. J. Environ. Manag..

[9] Gratani L., Crescente M.F., Varone L. (2015). Long-term monitoring of metal pollution by urban trees. Atmos. Environ..

[10] Loram A., Tratalos J., Warren P.H., Gaston K.J. (2007). Urban domestic gardens (X): The extent & structure of the resource in five major cities. Landsc. Ecol..

[11] Fujita A., Maeto K., Kagawa Y., Ito N. (2010). Effects of forest fragmentation on species richness and composition of ground beetles (Coleoptera: Carabidae and Brachinidae) in urban landscapes. Entomol. Sci..

[12] Carrete M., Tella J.L., Blanco G., Bertellotti M. (2009). Effects of habitat degradation on the abundance, richness and diversity of raptors across Neotropical biomes. Boil. Conserv..

[13] Zhou Z., Shao T., Wang P., Gao C., Xu Y., Guo E., Xu L., Ye Z., Peng X., Yu C. (2002). The Spatial Structures and the Dust Retention Effects of Green-land Types in the Workshop District of Wuhan Iron and Steel Com-pany. Acta Ecol. Sin..

[14] Liu Y., Guo J. (2009). The Research of NDVI-based Urban Green Space Landscape Pattern and Thermal Environment. Prog. Geogr..

[15] Shao T., Zhou Z., Wang P., Tang W., Liu X., Hu X. (2004). Relationship between urban green-land landscape patterns and air pollution in the central district of Yichangcity. Chin. J. Appl. Ecol..

[16] Feng L., Wang R. (2003). Evaluation, planning and prediction of ecosystem services of urban green space: A case study of Yangzhou City. Acta Ecol. Sin..

[17] Li F., Wang R.S. (2003). Method and Practice for Ecological Planning of Urban Green Space—Yangzhou City as the Case Study. Urban Environ. Urban Ecol..

[18] Iverson L.R., Cook E.A. (2000). Urban forest cover of the Chicago region and its relation to household density and income. Urban Ecosyst..

[19] Burgman M.A., Keith D., Hopper S.D., Widyatmoko D., Drill C. (2007). Threat syndromes and conservation of the Australian flora. Boil. Conserv..

[20] Palmer G.C., Fitzsimons J.A., Antos M.J., White J.G. (2008). Determinants of native avian richness in suburban remnant vegetation: Implications for conservation planning. Boil. Conserv..

[21] Costanza R., D’Arge R., Groot R.D., Farber S., Grasso M., Hannon B., Limburg K., Naeem S., O’neill R.V., Paruelo J. (1997). The value of the world’s ecosystem services and natural capital 1. Nature.

[22] Pimentel D., Wilson C., Mccullum C., Huang R., Dwen P., Flack J., Tran Q., Saltman T., Cliff B. (1997). Economic and Environmental Benefits of Biodiversity. Bioscience.

[23] Sutton P.C., Costanza R. (2002). Global estimates of market and non-market values derived from nighttime satellite imagery, land cover, and ecosystem service valuation. Ecol. Econ..

[24] Reid W.V., Mooney H.A., Cropper A., Capistrano D., Carpenter S.R., Chopra K., Dasgupta P., Dietz T., Duraiappah A.K., Hassan R. (2005). Millennium Ecosystem Assessment Synthesis Report. Millennium Ecosystem Assessment.

[25] Gren I.M., Groth K.H., Sylvén M. (1995). Economic Values of Danube Floodplains. J. Environ. Manag..

[26] Dixon J.A. (2000). Analysis and Management of Watersheds. The Environment and Emerging Development Issues: Volume 2.

[27] Pattanayak S.K. (2004). Valuing watershed services: Concepts and empirics from southeast Asia. Agric. Ecosyst. Environ..

[28] Turner R.K., van den Bergh J.C.J.M., Söderqvist T., Barendregt A., Van Der Straaten J., Maltby E., Van Ierland E.C. (2000). Ecological-economic analysis of wetlands: Scientific integration for management and policy. Ecol. Econ..

[29] Hanley N.D., Ruffell R.J. (1993). The contingent valuation of forest characteristics: Two experiments. J. Agric. Econ..

[30] Loomis J., Kent P., Strange L., Fausch K., Covich A. (2000). Measuring the total economic value of restoring ecosystem services in an impaired river basin: Results from a contingent valuation survey. Ecol. Econ..

[31] Lal P. (2003). Economic valuation of mangroves and decision-making in the Pacific. Ocean Coast. Manag..

[32] Jakobsson K., Dragun A.K. (1996). Contingent Valuation and Endangered Species: Methodological Issues and Applications.

[33] De Mendonça M.J., Sachsida A., Loureiro P.R. (2003). A study on the valuing of biodiversity: The case of three endangered species in Brazil. Ecol. Econ..

[34] Bandara R., Tisdell C. (2003). The net benefit of saving the Asian elephant: A policy and contingent valuation study. Ecol. Econ..

[35] Wu L., Lu J., Tong C., Liu C. (2003). Valuation of wetland ecosystem services in the Yangtze River estuary. Resour. Environ. Yangtze Basin.

[36] Xin K., Xiao D. (2002). Wetland Ecosystem Service Valuation—A Case Researches on Panjin Area. Acta Ecol. Sin..

[37] Xiao Y., Xie G., An K. (2003). Economic value of ecosystem services in Mangcuo Lake drainage basin. J. Appl. Ecol..

[38] Qiao X. (2003). Assessment on urban ecosystem services of guangzhou city. J. Beijing Norm. Univ..

[39] Yang Q., Lan C., Xin K. (2003). Evaluation on the value of the ecosystem services of the coastal zone in Guangdong and Hainan. Mar. Environ. Sci..

[40] Yu X., Xie G., Kai A. (2003). The function and economic value of soil conservation of ecosystems in Qinghai-Tibet Plateau. Acta Ecol. Sin..

[41] Ouyang Z.Y., Wang R.S. (1999). Ecosystem services and their economic valuation. Chin. J. Appl. Ecol..

[42] Ouyang Z., Wang X., Miao H. (1999). A primary study on Chinese terrestrial ecosystem services and their ecological-economic values. Acta Ecol. Sin..

[43] Chen Z.X., Zhang X.S. (2000). The value of ecosystem benefits in China. Chin. Sci. Bull..

[44] He H., Yang M., Pan Y., Zhu W. (2005). Measurement of terrestrial ecosystem service value in China based on remote sensing. Chin. J. Appl. Ecol..

[45] Bi X.L., Ge J.P. (2004). Evaluating Ecosystem Service Valuation in China Based on the IGBP Land Cover Datasets. J. Mt. Res..

[46] Xie G., Zhang Y., Lu C., Zheng D., Cheng S. (2001). Study on valuation of rangeland ecosystem services of China. J. Nat. Resour..

[47] Xie G.D., Lu C.X., Leng Y.F., Zheng D.U., Li S.C. (2003). Ecological assets valuation of the Tibetan Plateau. J. Natl. Resourc..

[48] Lu C., Xie G., Xiao Y., Yu Y. (2004). Ecosystem diversity and economic valuation of Qinghai-Tibet Plateau. Acta Ecol. Sin..

[49] Burley J.B. (1989). Multi-model habitat suitability index analysis in the Red River Valley. Landsc. Urban Plan..

[50] Burley J.B. (1991). A vegetation productivity equation for reclaiming surface mines in Clay County, Minnesota. Int. J. Surf. Min. Reclam. Environ..

[51] Loures L., Loures A., Nunes J., Panagopoulos T. (2015). The Green Revolution- converting post- industrial sites into urban parks—A case study analysis. Int. J. Surf. Min. Reclam. Environ..

[52] Cui C., Xu X. (2010). Relative Assessment of Green Space Ecosystem Service in Beijing Region. Acta Sci. Nat. Univ. Pekin..

[53] Feng T. (2016). Study on Agricultural Land Layout in Beijing Plain Area for Eco-friendly Target. Ph.D. Thesis.

[54] Zhu W., Pan Y., Zhang J. (2007). Estimation of net primary productivity of Chinese terrestrial vegetation based on remote sensing. J. Plant Ecol..

[55] Chen Z., Su X., Liu S., Zhang X. (1998). Study on Ecological Efficiency of Urban Landscape Architecture in Beijing (3). Chin. Landsc. Archit..

[56] National Environmental Protection Agency (1998). China’s Biodiversity: Country Study.

[57] Yang L. (2012). Studies on Water Consumption and Irrigation Model of Compound Plant Ecosystem in Urban Green Space in Beijing. Ph.D. Thesis.

[58] Cheng K., Cui G., Wang J., Li J. (2000). Evaluation on the economic value of the forest biodiversity in Labagoumen forest region. J. Beijing For. Univ..

[59] Wu W. (2011). Study on the Value Assessment of Urban Green Space Ecosystem Services in Hangzhou. Ph.D. Thesis.

[60] Wang X. (2015). The String Studies of North Beijing Suburb Forest Park Based on Spatial Data Analysis. Ph.D. Thesis.

[61] Luo Z. (2011). Towards Landmark Recognition Via Large Scale Social Mesdia Mining. Master’s Thesis.

[62] Xue Y. (2013). Strategy for Propagation of Tourism Destination Image in Picture-Sharing Networks—Taking Flickr for Example. Master’s Thesis.

[63] Cen X. (2016). Correlation Analysis and Optimization between Land Use Landscape Patterns and Ecosystem Service Values—A Case Study of South Coast of Hangzhou Bay. Ph.D. Thesis.

[64] Fragstats Help. http://222.28.119.57/cache/6/03/www.umass.edu/89aacb17a169768ca68ee14d9fcdbd93/fragstats.help.4.2.pdf.

[65] Tang Y., Shao Q., Cao W., Yang F., Liu L., Wu D., Zhou S. (2018). The Ecosystem Services and Its Spatial Variation at Countyscale in the Southern Guizhou Based on Physical Assessment Method. Sci. Geogr. Sin.-CA.

[66] Zhang M.Y., Wang K.L., Chen H.S., Zhang C.H., Liu H.Y., Yue Y.M., Fan F.D. (2009). Quantified evaluation and analysis of ecosystem services in Karst areas based on remote sensing. Acta Ecol. Sin..

